# Modeling of CCR5 Recognition by HIV-1 gp120: How the Viral Protein Exploits the Conformational Plasticity of the Coreceptor

**DOI:** 10.3390/v13071395

**Published:** 2021-07-18

**Authors:** Célien Jacquemard, Florian Koensgen, Philippe Colin, Bernard Lagane, Esther Kellenberger

**Affiliations:** 1 UMR 7200 CNRS Université de Strasbourg, 67400 Illkirch, France; jacquemard@unistra.fr (C.J.); koensgen.florian@gmail.com (F.K.); 2 Infinity, Université Toulouse, CNRS, INSERM, UPS, CEDEX 03, 31024 Toulouse, France; philippe.colin@inserm.fr

**Keywords:** viral entry, GPCR, flexibility, conformation, binding mode

## Abstract

The chemokine receptor CCR5 is a key player in HIV-1 infection. The cryo-EM 3D structure of HIV-1 envelope glycoprotein (Env) subunit gp120 in complex with CD4 and CCR5 has provided important structural insights into HIV-1/host cell interaction, yet it has not explained the signaling properties of Env nor the fact that CCR5 exists in distinct forms that show distinct Env binding properties. We used classical molecular dynamics and site-directed mutagenesis to characterize the CCR5 conformations stabilized by four gp120s, from laboratory-adapted and primary HIV-1 strains, and which were previously shown to bind differentially to distinct CCR5 forms and to exhibit distinct cellular tropisms. The comparative analysis of the simulated structures reveals that the different gp120s do indeed stabilize CCR5 in different conformational ensembles. They differentially reorient extracellular loops 2 and 3 of CCR5 and thus accessibility to the transmembrane binding cavity. They also reshape this cavity differently and give rise to different positions of intracellular ends of transmembrane helices 5, 6 and 7 of the receptor and of its third intracellular loop, which may in turn influence the G protein binding region differently. These results suggest that the binding of gp120s to CCR5 may have different functional outcomes, which could result in different properties for viruses.

## 1. Introduction

Entry of HIV-1 into the host cell (CD4 T lymphocytes, macrophages) is a complex and dynamic process that is mediated by its envelope glycoprotein Env. Env is composed of two non-covalently linked subunits, gp120 and gp41, which assemble as spike-shaped, symmetrical trimers on the virus surface. In the ligand-free state of the Env trimer, the three gp41 subunits are anchored into the viral membrane and lie at the bottom of the trimer, capped with gp120 subunits. The gp120s are exposed for the sequential and specific recognition of two membrane proteins of the host cell, the receptor CD4 and a chemokine receptor acting as coreceptor, either CC chemokine receptor type 5 (CCR5) or CXC chemokine receptor 4 (CXCR4). The binding of gp120 to cellular receptors brings the virus closer to the host cell and leads to exposure of gp41 that inserts into the host cell plasma membrane. Subunit gp41 refolds into a six-helix bundle (6HB), thereby triggering the fusion of viral and cell membranes and the release of the viral nucleocapsid into the cytoplasm [1].

**Figure 1:**
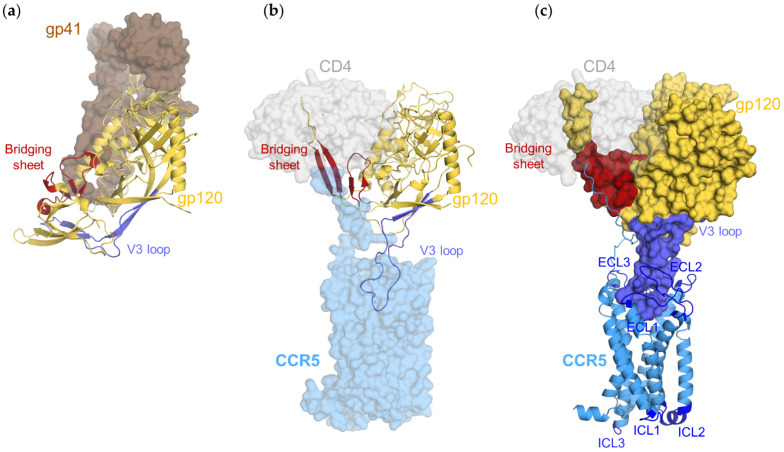
Structures of gp120 and its domains: (**a**) structure of closed form of gp120 (gold cartoon) in complex with gp41 (dark brown surface); (**b**) structure of open form of gp120 (gold cartoon) in complex with CD4 (gray transparent surface) and CCR5 (blue transparent surface); (**c**) structure of open form of gp120 (gold surface) in complex with CD4 (gray transparent surface) and CCR5 (blue cartoon). The gp120 V3 loop and the bridging sheet are highlighted in blue and red, respectively. The extracellular and intracellular loops of CCR5 are highlighted in dark blue. Image (**a**) prepared from PDB ID 5CEZ; Images (**b**,**c**) prepared from PDB ID 6MEO.

The role of Env in viral entry is closely related to its capacity to sample distinct conformational states [2]. Structural and biophysical studies on membrane-embedded [3,4,5,6] or soluble Env trimers [7,8] have shown that binding to CD4 is accompanied by the reorientation of each protomer of the trimer and restructuring of gp120 and gp41 subunits (Figure 1a,b and Figure S1a). In the ground, pre-fusion state, Env preferentially adopts a closed conformation, where gp41 and the binding sites to the coreceptor are shielded. Binding to CD4 stabilizes an open conformation of Env, which likely preexists in equilibrium with the closed one [9] and other open conformations [10]. Opening of the trimer involves outward movement and rotation of gp120 subunits, while the C-terminal part of gp41′s HR1 domain elongates to adopt a fusion-competent conformation. The gp120′s V1/V2 loops move from the trimer apex to the sides of the trimer, releasing the V3 loop, which contains determinants for binding the coreceptor. At the boundary between gp120’s inner and outer domains, movement of V1/V2 loops also triggers formation of the four-stranded bridging sheet domain (BS), which also contributes to coreceptor binding.

Several mutagenesis studies [11,12] and molecular modeling approaches [13,14,15,16] have provided information on the interaction of gp120 with the coreceptor. However, only very recently has an atomic-level description of this process, with the resolution by cryo-EM of the structure of a gp120 in complex with soluble CD4 (sCD4) and unmodified CCR5, been provided (Figure 1b,c) [17]. At this stage, gp120 has probably been shed from gp41, which then refolds to trigger fusion. In fact, the structure of the gp120 core does not change when the protein is bound to CD4 alone [7] nor together with CCR5 [17], suggesting that gp41 does not influence gp120 binding to CCR5, and conversely, that binding to CCR5 is not responsible for detachment of gp120 from gp41 [17]. Overall, the binding mode of gp120 to CCR5 resembles that of chemokines and involves two recognition sites [17]. The N-terminal domain of CCR5 adopts an extended conformation and comes in close contact with BS and the base of the V3 loop, thus forming a binding site overlapping the chemokine recognition site 1 (CRS1, involving CCR5 residues 16–18). The stem and the tip of V3 insert into a cavity located in the upper third of the seven transmembrane domains (TMs) of the receptor, a region where the antagonist chemokine analog, [5P7]CCL5, had also been localized [16] (chemokine recognition site 2, CRS2). While so doing, V3 folds into a conformation which, like that of the N-terminal domain of [5P7]CCL5, is capable of interacting with residues of all the helices forming the transmembrane cavity of CCR5, i.e., TMs 1–3 and 5–7, and also with the extracellular loop 2 (ECL2) of the receptor. Of note, unlike chemokines, the gp120 used in the cryo-EM structure (gp120 from the HIV-1 strain 92BR020, gp120_92BR020_) does not occupy the upper part of CRS2 nor the vicinal pivot between CRS1 and CRS2 (CRS1.5), a region comprising Pro19 and Cys20 and connected with extracellular loop 3 (ECL3) through the Cys20–Cys269 disulfide bridge. As a consequence, it has been suggested that different gp120s from other HIV-1 strains could differentially explore the CCR5 transmembrane cavity.

In the cryo-EM structure of the CD4–gp120–CCR5 complex, several observations led the authors to postulate that CCR5 adopts an inactive conformation. First, the arrangement of the TMs, especially TM6 that is critical for the activation of the class A G-protein coupled receptors (GPCRs) to which CCR5 belongs [18], does not differ between CCR5-bound gp120 [17] and CCR5 bound to the inverse agonist maraviroc (MVC) [19]. Also, heterotrimeric G proteins, which stabilize CCR5 in an active state [20], were not present in the CCR5–gp120 complex. Consistently with this, the authors also failed to observe G-protein dependent signaling of CCR5 in response to gp120–CD4 complexes [17]. This latter observation, however, is in sharp contrast with numerous studies indicating that HIV-1 gp120s can behave as agonists (for reviews see [21,22]), although differences in the nature of the signaling pathways induced by gp120s and chemokines were noted in some instances [23,24,25,26,27,28]. These differences in the signaling capacities between gp120_92BR020_ and other gp120s could indicate that they do not stabilize/recognize the same CCR5 conformations (although other likely possibilities can be considered, e.g., differences in the nature of the cells into which signaling is measured). This hypothesis is actually supported by our previous work showing that distinct primary gp120s in complex with sCD4 do not bind the same CCR5 subpopulations on cell lines and primary cells [29]. In this respect, gp120s resemble other CCR5 ligands (antibodies, chemokines) that also bind differentially to heterogeneous CCR5 subpopulations at the cell surface [30,31,32,33]. Heterogeneity of CCR5 relates to forms of the receptor that are differentially post-translationally modified (sulfated, O-glycosylated) [34,35] or exist in different oligomerization [29,36] or conformational states [20,31,37]. Our previous results also suggested that a link could exist between the nature of the CCR5 molecules to which gp120s bind, and phenotypic properties of the viruses, such as resistance to CCR5 entry inhibitors and cellular tropism [20,29,38,39]. We also postulated that differential binding of different gp120s to distinct CCR5 subpopulations could underlie the activation of distinct signaling pathways, which could help shape the properties of viruses. Whether the different CCR5 subpopulations that are differentially bound by distinct gp120s represent distinct conformations of the receptor remains, however, poorly known. And if so, it also remains to characterize the nature of these conformations and to what extent they differ from those induced by chemokines.

To address these issues here, we carried out molecular modeling and molecular dynamics simulations to study the molecular determinants and the conformational consequences of the interaction between CCR5 and four distinct gp120s in complex with CD4. Results indicate that differences in the sequence of gp120 translate into distinct modes of binding to the coreceptor. This is revealed by specific conformational changes in the gp120 variable loops, as well as by differences in the configuration of the binding pocket of CCR5 and the arrangement of its extracellular and intracellular loops and of its TMs ends. Interestingly, specific movements of the intracellular ends of TM5–7 in the gp120–CCR5 complexes result in a position of intracellular loop 3 (ICL3) that diverges from its position in ligand-free CCR5. Our observations that gp120s impose specific conformational signatures in CCR5 leave open the possibility that they could have distinct functional outcomes upon coreceptor engagement.

## 2. Materials and Methods

### 2.1. Homology Modeling

The three-dimensional structures of ligand-free CCR5 (CCR5_free_), and of the CD4–gp120_#25_–CCR5, CD4–gp120_#34_–CCR5, CD4–gp120_Bx08_–CCR5 and CD4–gp120_JR-FL_–CCR5 complexes, were built from the cryo-EM structure of CD4–gp120_92BR020_–CCR5 (PDB entry: 6MEO) [17].

The sequences of CCR5 and CD4, which are native in the template, were not modified. The CCR5 structure (Uniprot accession number: P51681) is complete from the first residue (Met1) to the end of helix 8 (Gln313). The CD4 structure (Uniprot accession number: P01730) covers the Ig-like domains D1 and D2 (from Lys26 to Val201).

The structure of gp120_#25_, gp120_#34_ [29], gp120_Bx08_ (accession number in HIV sequence database of Los Alamos National Laboratory: AY713411) and gp120_JR-FL_ (accession number in HIV sequence database of Los Alamos National Laboratory: U63632) were modeled by homology to gp120_92BR020_ (Glu32 to Gly495), except for the missing variable domains V1, V2 (Asp133 to Asn186) and V4 (Gly404 to Ser411), which were modeled from the low resolution cryo-EM model of the open trimer of CD4–gp120–gp41, stabilized by the 17b antibody (PDB entry: 3J70). In practice, the coordinates of the residues Val127 to Ser195 (V1/V2) and Asn386 to Pro417 (V4) in the PDB file 3J70 were inserted into the gp120 structure in the PDB 6MEO file using the loop grafting tool MOE 2018.01, yielding a complete template suitable for building models using the Protein Align/Superimpose and Homology Model tools (default options, best model) of the MOE 2018.01 software. 

The protonation state was predicted with the Protonate3D tool (default options) of the MOE 2018.01 software. 

Of note, N-glycans attached to gp120_92BR020_ in the cryo-EM structure were not transferred into models. As the Asn residues were not strictly conserved in the four gp120s studied, we avoided having to distinguish the direct effects of mutations on the complex conformation and their indirect effects mediated by N-glycans.

Point mutations T177A, Y187A, D276A and E283Q were each introduced to CD4–gp120_#25_–CCR5 and CD4–gp120_#34_–CCR5 with the Protein Builder tool of MOE version 2018.01, yielding eight additional models. 

### 2.2. Molecular Dynamics Simulation

The thirteen models were prepared using the CHARMM-GUI web service version 2.1 (CCR5–gp120_#25_–CD4, CCR5–gp120_#34_–CD4) and version 3.0 (CCR5_free_, CCR5–gp120_Bx08_–CD4 and CCR5–gp120_JR-FL_–CD4 and the eight mutants) [40]. The CCR5 TMs were inserted into a lipid bilayer using the Bilayer Builder tool [41], fixing the orientation of CCR5 so as to place helix 8 at the lipid/water interface. The lipid bilayer is composed of phosphatidylcholine, phosphatidylethanolamine and cholesterol in a 2:2:1 ratio in its upper and lower parts. The cubic box length was to set to a = b = 200 Å. The hydration layer had a thickness of 22.5 Å on both sides of the bilayer of the *z*-axis, for a total height of ≈175 Å. K^+^ and Cl^−^ ions were added at a final concentration of 0.15 M. They were placed by the Monte-Carlo method.

CHARMM-GUI places the proteins such a way that the principal axes are aligned with those of the box. As the CD4–gp120–CCR5 complex has roughly a T-shape, a large volume of the box contained only water. The size of the system was therefore reduced by aligning the 2^nd^ principal axis with the small diagonal of the box (shrinkbox.py, Supplementary Materials). The lengths were thus decreased to a = b ≈ 120 Å.

The shrunk system was processed by the charmmlipid2amber.py v.2.0.3 script (available on the CHARMM-GUI website) to rename lipid, water and ion residues according to AMBER naming convention. Histidine residues were corrected according to the AMBER naming convention. Tyr10 and Tyr14 residues of CCR5 were modified to sulfotyrosine with the Protein Builder tool of MOE version 2018.01. Cysteine’s name was modified to CYX and the hydrogen atoms of the corresponding sulfhydryl group removed if the S–S distance between two Cys was lower than 2.5 Å (fix_SSbridge.py, Supplementary Materials). Two disulfide bridges were defined in CCR5: between TM3 and ECL2 (residues 101 and 178) and between the N-terminal domain and ECL3 (residues 20 and 269). Gp120 has eight disulfides bridges: residues 54–74, 119–205, 126–196, 218–247, 228–239, 296–331, 378–445 and 385–418. Finally, two disulfide bridges were defined in CD4: residues 41–109 and 155–184. At this stage, a visual inspection ensured that no aberrations were present in the structure (e.g., interleaved rings).

The topology and coordinate files were generated using the tleap program of the AMBER16 suite and using the force field parameters ff14SB for proteins, lipid14 for the lipid bilayer, TIP3 for water molecules [42,43] and previously defined for the sulfotyrosine residue [39]. The system charges were neutralized, if necessary, by adding K^+^ or Cl^−^ counter ions.

The simulations were performed on a farm of graphics processors (GPU Nvidia^®^ Tesla K80 and V100) with the pmemd.cuda program from the AMBER16 suite and the CUDA 8.0 library (K80, for the four native CCR5 complexes) or the AMBER18 suite and the CUDA 10.1 library (V100 for the eight mutant complexes and the ligand-free CCR5) [44,45]. Each system was first minimized in 15,000 steps using the steepest-descent method for the first 10,000 steps and the conjugate gradient method for the next 5,000 steps. Then, it was heated from 0 to 300 K by applying the Langevin thermostat over a period of 75 ps at constant volume by restraining the atomic coordinates using harmonic potentials. The restraints were gradually released for 175 ps at constant volume (Table S1). The system was equilibrated for 10 ns (except CCR5_free_: 30 ns) at constant pressure. For the production stage, five independent runs were produced over a period of 100 ns by randomly changing the seed of the Langevin thermostat at each stage. The length of a run was adjusted so as to stop simulation before a possible interaction between gp120 or CD4 and the lipid bilayer.

### 2.3. Definition of Protein Domains

The gp120 numbering scheme refers to the Env protein sequence of HxB2 virus (Uniprot accession number: P04578) (Figure S1). The gp120 domains are defined by the HIV sequence database at Los Alamos National Laboratory (www.hiv.lanl.gov (accessed on 21 May 2019)) as follows: V1: 130–157; V2: 156–196; V3: 294–332; V4: 385–418; V5: 459–466. The gp120 core excludes the five variable loops and the N-terminal and C-terminal domains (C1: 74–129; C2: 197–293; C3: 333–384; C4: 419–458; C5: 467–490).

CCR5 is made of seven TMs linked by three extracellular and three intracellular loops. Domain definition was based on the position of the residues with respect to the lipid bilayer. A TM is hence fully included between the outer and inner planes of the lipid bilayer. The CCR5 TMs used throughout the manuscript correspond to TM1: 31–57; TM2: 64–88; TM3: 99–129; TM4: 143–164; TM5: 190–219; TM6: 235–256; TM7: 277–299. 

### 2.4. Trajectory Analysis

#### 2.4.1. Deviation of the Atomic Coordinates of the Main Chain of Proteins

The deviation of the atomic coordinates during the equilibration and production was estimated by the root-mean-square deviation (RMSD, Equation 1) using the rmsd command of the CPPTRAJ software version 17.00 [46] for the Cα, C, O and N backbone atoms, after the best-fit alignment of the protein or the protein domain:

(1) $${\mathrm{RMSD}}_{t}=\sqrt{\frac{1}{N}\overset{N}{\underset{n=1}{\sum }}{\left(x{\left(\min\right)}_{n}-x_{n}\right)}^{2}}$$

where:

- RMSD*_t_*—the RMSD of the frame *t*
- min—the minimized structure, taken as reference
- *x*—the position of the atom *n*
- *N*—the total number of atoms in the set.

#### 2.4.2. Fluctuation of Atomic Coordinates Per Residue

The atomic fluctuations (RMSF) during the production stage were calculated on Cα atoms after 3D alignment of CCR5 TMs, gp120 core or CD4 onto the first structure of the production. It is estimated by the root-mean-square fluctuation (RMSF, Equation (2)) using a Python script with MDAnalysis module v.0.20.1:

(2) $${\mathrm{RMSF}}_{p,r}=\sqrt{\frac{1}{T}\overset{T}{\underset{t=1}{\sum }}{\left(x{\left(\mathrm{avg}\right)}_{t}-x_{t}\right)}^{2}}$$

where:

- RMSF*p,r*—the RMSF for the residue *r* of the protein *p*
- avg—the averaged structure
- *x*—the position of the atom
- *T*—the total number of structures

#### 2.4.3. Clustering of Structures

The structures of the production stage were clustered by a hierarchical agglomerative algorithm using RMSD as a measure of distance and an average linkage. A total of five representative frames were selected per trajectory using the cluster command of CPPTRAJ version 17.00 with default options. Beforehand, all structures were aligned onto the seven transmembrane helices of CCR5 in the minimized structure of the CD4–gp120_#25_–CCR5 complex using the rmsd command of CPPTRAJ version 17.00 with default options (helix 1: 22–58; helix 2: 63–92; helix 3: 97–132; helix 4: 141–167; helix 5: 186–224; helix 6: 228–265; helix 7: 268–300; definition from gpcrdb.org/protein/ccr5_human/, accessed on 21 May 2019) [47].

#### 2.4.4. Projection of CCR5 Extracellular and Intracellular Helix Extremities

All the structures were aligned onto the TMs of CCR5 of the same reference structure (see above). For each system, the seven helices were redefined by shrinking so that the secondary structure of the first and the last residues was a helix-α according to the DSSP algorithm throughout more than half of the simulation time (helix 1: 26–57; helix 2: 64–89; helix 3: 98–131; helix 4: 142–165; helix 5: 187–223; helix 6: 228–259; helix 7: 269–300). The *x* and *y* centroid coordinates of the Cα atoms of the three extracellular (or intracellular) terminal residues of a helix were projected onto the outer or inner plane of the lipid bilayer, respectively. Contours delimit 90% of the density obtained by projecting the helix in all frames, based on a normal kernel density estimation. A standalone program called ATOLL, dedicated to producing projection images from trajectory files, is freely available in the repository (https://github.com/LIT-CCM-lab/ATOLL).

#### 2.4.5. Matrix of Structural Similarities

Local structural conservation in CCR5 was evaluated by all-against-all comparison of one in ten structures issued by the simulation of a complex or by the systematic pairwise comparison of one in ten structures issued by the simulation of a complex with that issued by the simulation of another complex. For each pair, the two compared structures were aligned onto CCR5 TMs. Comparison allowed computing of RMSD matrix from Cα coordinates as follows (Equation (3)):

(3) $${\mathrm{RMSD}}_{t1,t2,d}=\sqrt{\frac{1}{N}\overset{N}{\underset{n=1}{\sum }}{\left(x_{1,n}-x_{2,n}\right)}^{2}}$$

where:

- *t*1 and *t*2—the frame of the simulated systems 1 and 2, respectively
- *d*—the analyzed CCR5 domain
- *N*—the total number of atoms in *d*
- *x*—the position of the atom *n*

The definition of domains is ICL1: 58–63; ECL1: 90–97; ICL2: 132–141; ECL2: 166–186; ICL3: 224–227 and ECL3: 260–268. Extra and intracellular loops correspond to the combination of previous definition. TMs are as defined above.

#### 2.4.6. Frequency Mapping of Non-Covalent Intermolecular Interactions

One in ten structures was taken from the simulated trajectories to sample intermolecular interactions. For each structure, all interatomic distances were calculated using the MDAnalysis module v.0.20.1. Hydrogen bonds were detected based on distance and angle, and ionic bonds based only on distance [48]. The hydrogen bonding frequency during the simulation was calculated for each relevant pair of residues taking into account the presence or absence of hydrogen bond between the two residues, regardless of the number of hydrogen bonds formed.

#### 2.4.7. Principal Component Analysis 

Principal component analysis (PCA) was used to quantify variations between the trajectories of the same system. All structures (five systems, five runs) were superimposed onto the initial minimized structure of CD4–gp120_#25_–CCR5 for the best fit of the Cα of CCR5 TMs to calculate an average structure of CCR5. Then all the structures were 3D-aligned onto the TMs of CCR5 average structure. The covariance matrix and its diagonalization were carried out using the commands “matrix covar” and “diagmatrix”, respectively, of CPPTRAJ v.17.00. The first 30 eigenvectors were calculated.

### 2.5. Binding Experiments to Wild-Type CCR5 and CCR5 Mutants

The binding experiments of ^125^I-CCL3 or ^35^S-gp120 complexed with sCD4 were carried out on crude membrane preparations from HEK 293T cells expressing wild-type or mutated CCR5 receptors (see below for details of these experiments). HEK 293T cells were cultured in DMEM (w/4.5 g/L glucose, L-Gln and sodium pyruvate) supplemented with 10% FCS, 100 μg/mL streptomycin and 100 units/mL penicillin. These cells were transiently transfected with pcDNA3.1 plasmids containing the sequences of the receptors using the PEI method, as described [49]. Forty-eight hours post-transfection, membranes were prepared as described previously [50] and receptor expression levels were determined by flow cytometry analysis. To this end, cells were stained with the anti-CCR5 mAbs CTC5 (R&D Systems, Minneapolis, MN, USA), 2D7 (BD Biosciences, Allschwil, BL, Switzerland) or 45531 (R&D Systems) and then with AlexaFluor 647-conjugated goat anti-mouse IgG (Invitrogen, Waltham, MA, USA), as described [29]. Data were acquired using a BD LSR-Fortessa flow cytometer (BD Biosciences) and analyzed using FlowJo Software. 

The binding experiments of ^125^I-CCL3 (0.2 nM) or ^35^S-labeled gp120s (10 nM, in the presence of 400 nM sCD4) on 1–2 µg or 20 µg of membrane proteins, respectively, were carried out as described in our previous works [51,52]. Briefly, membranes were incubated with the radioactive proteins at room temperature for 90 min in 0.1 mL final volume of assay buffer (50 mM Hepes, pH 7.4, 1 mM CaCl2, 5 mM MgCl2 and 0.5% BSA). Non-specific binding was measured on wild-type CCR5-expressing membranes in the presence of 10 µM maraviroc (Sigma-Aldrich, St. Louis, MI, USA) or using membranes from non-transfected cells, with similar results. Unbound radioligands were removed by filtering membranes through GF/B filters presoaked in 1% BSA (CCL3) or by centrifugation (gp120) and subsequent washing steps. Bound radioactivity was then measured using a Wallac 1450 Microbeta Trilux (PerkinElmer Life Sciences, Waltham, MA, USA) or a gamma counter (multi-crystal LB 2111 gamma counter, Berthold Technologies, Bad Wilbad, Germany). Radioactive gp120s and soluble CD4 were produced and purify as described in references [52,53], respectively.

## 3. Results

### 3.1. General Description of the Modeling and Molecular Dynamics Simulations

The four gp120s for which we modeled interaction with CCR5 are derived from the HIV-1 strains Bx08, JR-FL, #25 and #34. These gp120s display distinct cellular tropism (T-cell- vs macrophage-tropic) and differ in the nature and the number of the CCR5 molecules they recognize at the cell surface, as described in our recent study [29]. They show high sequence similarity and sequence identity ranging from 76 to 85%. Differences are manifested by mutations, insertions and deletions that occur mostly in the variable loops, in particular coreceptor binding regions (Figure S1).

Atomic-resolution models of these gp120s bound to CD4 and CCR5 were built by homology using the cryo-EM structure of the CD4–gp120_92BR020_–CCR5 complex (Figure S2). In this structure, CCR5 is in its native form, especially with a complete N-terminal domain showing sulfation of Tyr10 and Tyr14 known to control chemokine binding and HIV-1 entry [34,54]. For the purpose of the structural study, CD4 was truncated to its soluble form containing the four immunoglobulin-like domains (D1–D4), of which D1 is in contact with gp120. In our modeling, only the first two domains, D1 and D2, which were more visible in the structure and are sufficient to expose the coreceptor binding sites [55] and to promote HIV-1 entry [56], have been taken into account. Gp120_92BR020_ shares high sequence identity with the four gp120s studied here, gp120_Bx08_, gp120_JR-FL_, gp120_#25_ and gp120_#34_ (78 to 84%) (Figure S1c). Gp120_92BR020_’s structure misses the variable loops V1, V2 and V4, so these parts of the protein were modeled using another structure as template [57].

In our 3D models of CD4–gp120–CCR5 complexes, CCR5 is embedded in a hydrated lipid bilayer. Each CD4–gp120–CCR5 complex was submitted to five independent simulations by molecular dynamics simulations. Each simulation lasted 100 ns, a length sufficient to explore stable structures close to the initial state, which is common to all the models. We also simulated ligand-free CCR5 under the same conditions for reference.

### 3.2. CD4–gp120–CCR5 Is a Flexible Complex

**Figure 2:**
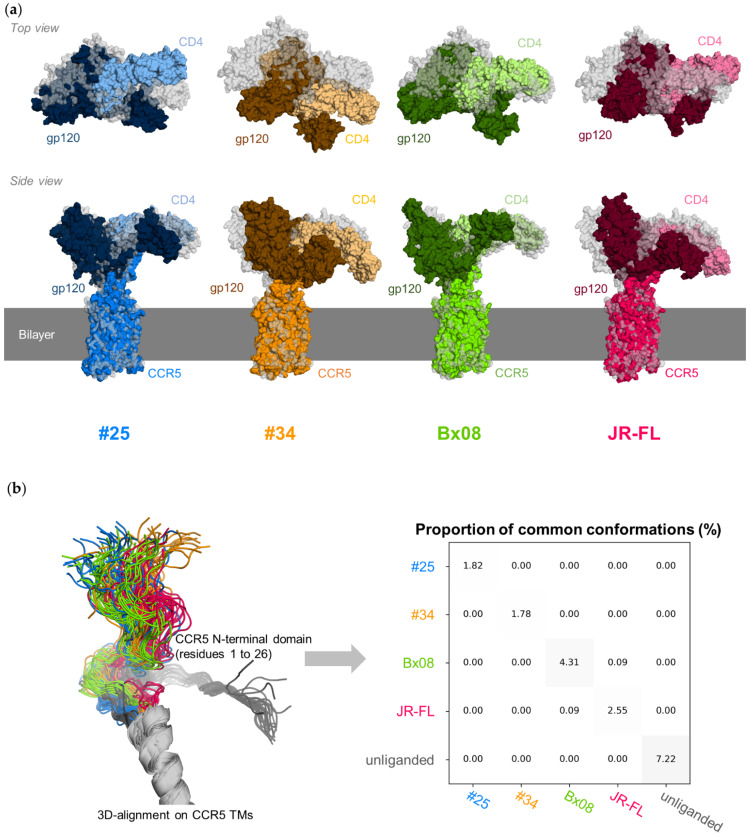
Overall structure of CD4–gp120–CCR5 complexes. The gp120 variants are colored as follows: #25 in blue, #34 in orange, Bx08 in green and JR-FL in red. All structures were 3D aligned onto CCR5 transmembrane residues: (**a**) last structure of a trajectory. CCR5, gp120 and CD4 are represented by molecular surfaces, colored using normal, dark and light tones, respectively. For the sake of comparison, the cryo-EM structure (PDB ID: 6MEO) is represented too, in grey; (**b**) diversity of the positioning of the N-terminal domain of CCR5 with respect to the TM inserted into the lipid bilayer. The N-terminal domain of ligand-free CCR5 is shown in grey. The proportion of common conformations is computed from the all-against-all comparison of one in ten snapshots issued from the simulation of the same complex (diagonal of the matrix) or the systematic pairwise comparison of one in ten snapshots issued during the simulation of two different complexes (off-diagonal terms of the matrix). The N-terminal conformation is common to two snapshots whether the deviation of the N-terminal Cα coordinates is low (RMSD < 2 Å).

During the simulation, the 3D-structure of the core of the three proteins in the complexes, each considered separately, does not deviate much from the structure of their cryo-EM template (median RMSD < 1.5 Å, <2.0 Å and <2.1 Å for CCR5 TMs, the gp120 core and CD4 D1 and D2, respectively) (Figure S3). The overall shapes of the CD4–gp120–CCR5 complexes, however, evolve significantly. Gp120, while initially straightly aligned with the CCR5 TMs, tilts towards the membrane (Figure 2a). This movement involves the bending of a hinge region encompassing the base of the gp120 V3 loop and the bound part of the CCR5 N-terminal domain.

Overall, gp120_#34_ experiences the greater movement, as compared to the other gp120s (Figure S3, see the plots labeled “All”). Nevertheless, the final position of gp120 relative to the CCR5 TMs is different at the end of each run. The N-terminal domain of CCR5 moves in multiple directions. Its position with respect to the TMs of CCR5 is not preserved during the simulation of the same complex (Figure 2b). Moreover, the positions explored by the N-terminal domain of CCR5 are different in the four complexes, as well as in the ligand-free receptor, but we cannot exclude that this observation is due to insufficient sampling. Overall, the results suggest that distinct positions of the gp120 core can allow binding to CCR5. However, the movements of gp120 could be more constrained in a context where the protein interacts with membrane anchored-CD4.

During the reorientation of gp120 relative to the CCR5 TMs, the structure of the constant domains of the gp120s does not change (Figures S3 and S4). In sharp contrast, the five variable loops protruding from the gp120 core experience considerable atomic fluctuations. The fluctuations are greater in V1 and V2 than in the other loops (Figure S4). Consistent with its larger overall reorientation relative to CCR5 TMs, gp120_#34_ exhibits the largest fluctuations of V1 and V2 loops. In CCR5, the intracellular and extracellular loops are also more flexible that the TMs core, yet to a lesser extent than gp120 loops. The maximal fluctuation per residue is around 3.5 Å in CCR5, as in extracellular loop 3 (ECL3) of the receptor complexed with gp120_JR-FL_, and greater than 10 Å in gp120, as in gp120_#34_’s V1 (Figure S4). On the whole, the pattern of the atomic fluctuations along the sequences of gp120 and CCR5 varies considerably between the four complexes. This suggests that differences in the sequence of gp120 translate into specific conformational changes in CCR5. The PCA carried out on the TMs and loops of CCR5 distinguishes the five systems studied while grouping the data for the five repetitions of each (Figure S5). The quantitative and qualitative characterization of these differences is presented in the following paragraphs.

### 3.3. Gp120s Differentially Shape the Extracellular Side of CCR5

**Figure 3:**
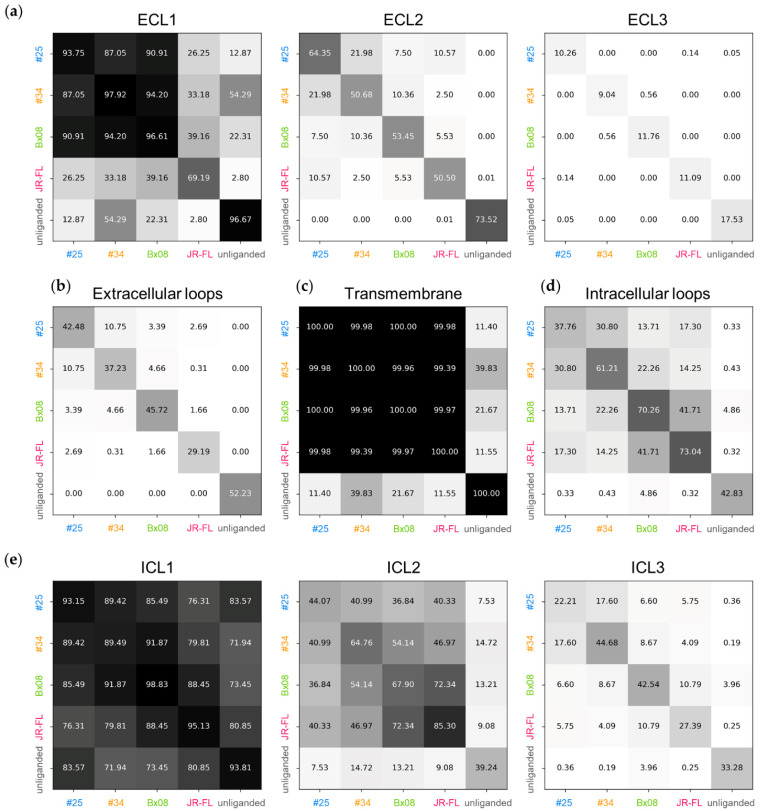
Percent of common positions of the CCR5 loops and 7-TMs: (**a**) ECL1, ECL2 and ECL3; (**b**) the three ECLs; (**c**) 7TMs; (**d**) the three ICLs; (**e**) ICL1, ICL2 and ICL3; (**a**–**e**) all structures were 3D-aligned on the input coordinates of CCR5 TMs. The proportion of common conformations is computed from the all-against-all comparison of one in ten snapshots issued from the simulation of the same complex (diagonal of the matrix) or the systematic pairwise comparison of one in ten snapshots issued during the simulation of two different complexes (off-diagonal terms of the matrix). Position is common to two snapshots if the deviation of the Cα coordinates of the domain is low (RMSD < 2 Å).

After having observed that the position of the N-terminal domain of CCR5 is not characteristic of binding to gp120, we next examined whether any structural signatures are present in the CCR5 extracellular loops ECL1, ECL2 and ECL3, by considering the fluctuation of residues (Figure S4), the average deviation of coordinates (Figure S6), the percent of common positions (Figure 3) and the distribution of characteristic distances (Figure S7). The position of ECL1 is common to three complexes, CD4–gp120_#25_–CCR5, CD4–gp120_#34_–CCR5 and CD4–gp120_Bx08_–CCR5 (average RMSD < 1.5 Å, within a complex or between two complexes). It is slightly more fluctuating in CD4–gp120_JR-FL_–CCR5 (average RMSD = 1.7 Å) and deviates from the one observed in the three other complexes (average RMSD > 2.4 Å). By comparison, ECL1 is fairly rigid in the ligand-free receptor (average RMSD = 1.2 Å) yet its position, on the whole, differs from those observed in the complexes (average RMSD range from 2 Å to 3.3 Å). The β-hairpin structure of ECL2 is well preserved, yet its tilt towards the transmembrane cavity center varies (Figure S7). A significant part of the explored positions is however common to two or more CD4–gp120–CCR5 complexes (e.g., ≈22% of positions were common between CD4–gp120_#25_–CCR5 and CD4–gp120_#34_–CCR5). Note that none of the ECL2 positions observed in the four complexes are nevertheless found in the structures of the ligand-free CCR5 (average RMSD > 3.9 Å). The structural differences between the four CD4–gp120–CCR5 complexes are even more noticeable for ECL3. This loop is more oriented towards the membrane lipids in CCR5 bound to gp120_#34_ or gp120_Bx08_, while it tends to rise above the receptor in CCR5 bound to gp120_JF-RL_ and even more to gp120_#25_ or if ligand-free (Figure S7). Due to their size and solvent exposure, ECL2 and ECL3 are expected to modulate accessibility of the ligand to CRS2. In all the simulations, ECL2 and ECL3 remain mobile, in a pendulum movement of large amplitude (up to 6 Å). This indicates that each gp120 can recognize a variety of CCR5 forms that differ in the positions of ECL2 and ECL3. The nature of the positions that ECLs adopt, however, markedly differ between the four gp120s.

**Figure 4:**
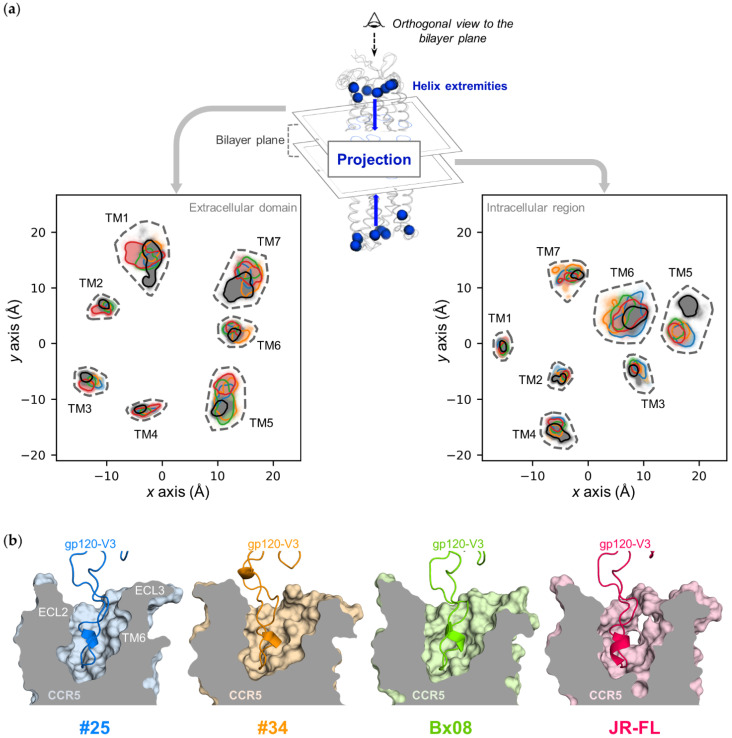
CCR5 transmembrane cavities: (**a**) projection of TMs ends from intracellular (left) and extracellular (right) domains; (**b**) 3D view of clipped CCR5 TM cavity (surface) bound to gp120 V3 loop (cartoon); (**a**,**b**) Data points and protein structures of CD4–gp120_#25_–CCR5, CD4–gp120_#34_–CCR5, CD4–gp120_Bx08_–CCR5, CD4–gp120_JR-FL_–CCR5 and ligand-free CCR5 are colored in blue, orange, green, red and grey, respectively.

Although the overall 7-TMs structure is not changed upon simulation (Figure 3 and Figure S6c), we wondered whether conformational changes of the ECLs could impact the ends of the CCR5 TMs. By plotting the ends of the CCR5 TMs from above the plane of the membrane, we observed that the position of the extracellular ends of the TMs indeed depend on the nature of the gp120 (Figure 4a, left panel). In CD4–gp120_JR-FL_–CCR5, the extracellular ends of TM1, TM2 and TM3 accentuate their tilt towards the membrane lipids, widening one half of the transmembrane cavity. In addition, TM1 undergoes large motion as compared with the other TMs. In CD4–gp120_#34_–CCR5, the extracellular ends of TM5, TM6 and TM7 tend to lean towards the membrane lipids, widening the opposite part of the transmembrane cavity. In CD4–gp120_Bx08_–CCR5, only the extracellular end of TM5 shows such a marked behavior. In CD4–gp120_#25_–CCR5, the extracellular ends of TMs disperse less around their initial position than in the three other complexes, and also than in the ligand-free receptor. Although each exhibit specificities in the positioning of the ends of their TMs, the four complexes have some points in common compared to the ligand-free receptor. The major difference concerns TM1 and TM7, at the level of CRS1.5, which suggests that the constraints imposed by the binding of gp120 to the CSR1 and CSR2 sites have an impact on this region that is not in direct interaction with gp120. The positioning of the TM5 end also distinguishes ligand-free CCR5. It is either completely different from that observed in a complex (CD4–gp120_#25_–CCR5, CD4–gp120_JR-FL_–CCR5), or else covers only a small part of the space explored in a complex (CD4–gp120_#34_–CCR5, CD4–gp120_Bx08_–CCR5). 

Considered altogether, these data show that the gp120s, by modulating the conformations of ECLs and of the extracellular ends of TMs, shape differently the accessibility to the transmembrane cavity, and therefore to CRS2. While the cavity opening is enlarged in CCR5–gp120#_34_, it is narrower in CCR5–gp120_#25_, and in between for the other complexes (Figure 4b). The pairwise comparisons of molecular dynamics snapshots, focusing on the extracellular side of CCR5 as a whole, actually suggest very little overlap in the conformations adopted by the receptor between the four gp120–CCR5 complexes (Figure 3b). This may explain why different HIV-1 strains/Envs do not recognize the same CCR5 conformations at the surface of HIV-1 target cells [29,30].

### 3.4. Gp120s Differentially Shape the Intracellular Side of CCR5

We also analyzed whether the gp120s influence the position of the intracellular ends of the TMs (Figure 4a, right panel). In the four complexes, as well as in the ligand-free receptor, the ends of the TM1–4 each have a well-defined position, while the ends of the TM5–7 have positions which vary during the simulation. As in its extracellular part, the 7-helices bundle of ligand-free CCR5 differs from that of the four complexes by a characteristic positioning of TM5 further from the center of the TMs. Comparison of the four complexes does not reveal much difference in the position of the intracellular ends of the TMs, but nevertheless the space explored by TM6 and TM7 varies subtly depending on gp120. CD4–gp120_#25_–CCR5 shows the greatest flexibility of TM6 end, while in the CD4–gp120_#34_–CCR5 complex, TM6 get closer to TM7. In addition, CD4–gp120_#25_–CCR5 and CD4–gp120_#34_–CCR5 each have their own positions of the TM7 end. 

Since the ends of TM6 and TM7 are linked by ICL3, we assumed that the intracellular loops may have sensed the allosteric effect of the binding of gp120. We continued our analysis of CCR5 conformation by considering the average deviation of coordinates (Figure S6) and the percent of common positions (Figure 3d,e). ICL1 conformation is highly conserved (RMSD < 1.3 Å in the simulation of a complex or of the ligand-free receptor and RMSD < 1.8 Å between them). ICL2 is more flexible (1.4 < RMSD < 2.3 Å in the simulation of a complex or of the ligand-free receptor), with a marked difference in the conformation of ligand-free and gp120-bound CCR5 (RMSD > 2.8 Å). As expected from the projection plot of TM ends, ICL3 is even more flexible (2.4 < RMSD < 3.4 Å in the simulation of a complex or of the ligand-free receptor), with hardly no conformations common to two complexes (at max. 17% between CD4–gp120_#25_–CCR5 and CD4–gp120_#34_–CCR5).

In summary, simulations have shown that the conformational populations targeted by the different gp120s also differ in the intracellular portion of the receptor, providing a structural basis for explaining the functional differences of viral proteins in signaling.

### 3.5. Gp120s Show Similar yet Different Binding Modes to CCR5

**Figure 5:**
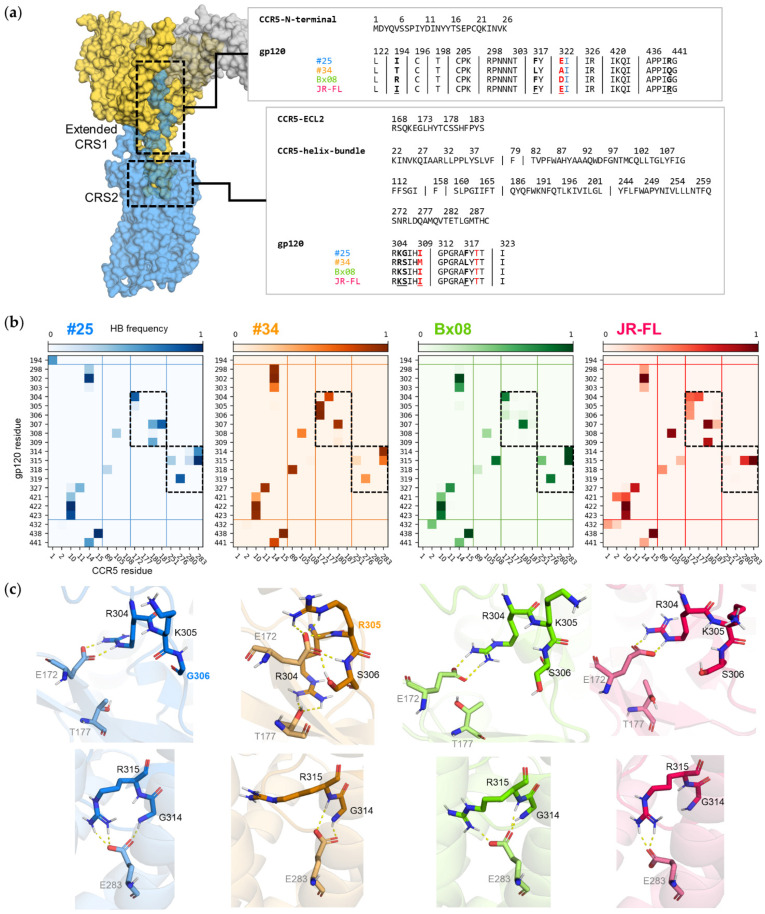
CCR5/gp120 interfaces in CD4–gp120–CD4 complexes: (**a**) amino acid sequences. Gp120 residues in red form interaction with CCR5 in the simulated structure but not in the cryo-EM structure (PDB ID: 6MEO). Gp120 residue Ile322A, in blue, is an insertion as compared with HxB2 reference sequence. Underlined positions are non-conserved residues; (**b**) frequency of the hydrogen bonds formed between gp120 and CCR5 in simulated structures. Frequency ranges from 0 (light color) to 1 (dark color). Lines delineate protein domains (gp120: bridging sheet, V3, V2; CCR5: N-terminal domain, helix 2–3 minor subpocket; ECL2 and top of helix 5; helix 6–7 major subpocket) and boxes focus on contacts shown on panel (**c**); (**c**) key interactions between gp120 V3 and CCR5 TM (top) and ECL2 (bottom). CCR5 is shown as ribbon, residues in interaction as sticks; (**b**,**c**) data points and protein structures of CD4–gp120_#25_–CCR5, CD4–gp120_#34_–CCR5, CD4–gp120_Bx08_–CCR5 and CD4–gp120_JR-FL_–CCR5 are colored in blue, orange, green and red, respectively. In (**c**), labels of gp120-specific amino acids are colored according to the same scheme.

The interface between CCR5 and gp120 in CRS1, which is extended in simulated CD4–gp120–CCR5 complexes as compared to the cryo-EM structure, involves the first 26 amino acids of the CCR5 N-terminal domain and 29 residues in the bridging sheet and the V3 base of gp120 (Figure 5a). Only four positions vary in sequence between the four studied gp120s (positions 194 in V2, 317 and 322 in V3 and 440 in the fourth conserved domain, C4). In the four simulated CD4–gp120–CCR5 complexes as well as in the cryo-EM structure [17], the two sulfated residues of CCR5, Tys10 and Tys14, each form an ionic bond with gp120, respectively in the bridging sheet (Lys421 of C4) and in the base of V3 (Arg298). The local environments of both tyrosine residues is preserved in the four complexes (Figure S8). In particular, throughout all simulations, they form efficient hydrogen bonds with the backbone atoms of the gp120 residues Gln422 (C4), Ile423 (C4) and Asn302 (V3 base). Overall, the patterns of the hydrogen bonds between the CCR5 N-terminal domain and gp120 are very similar in the four CD4–gp120–CCR5 complexes (Figure 5b). While organization of the first interface is thus globally similar, subtle structural differences are however revealed by the simulations. For example, Arg298 can form two hydrogen bonds with Tyr14 in addition to the ionic bond mentioned above, yet mainly in the CD4–gp120_#34_–CCR5 complex. Another example is Ile194 (V2 stem), which forms hydrogen bonds with CCR5 Met1 exclusively in the CD4–gp120_#25_–CCR5 complex.

The interface between CCR5 and gp120 in CRS2 involves CCR5 ECL2 binding to gp120’s V3 stem and the CCR5 transmembrane cavity binding to gp120’s V3 tip. Again, the corresponding gp120 sequences are well conserved, with variation in only 4 of 16 positions (305, 306, 309 and 317), and no dramatic changes in the residue size, charge or polarity (Figure 5a). Nevertheless, the simulations reveal distinctive binding patterns between the gp120s and CCR5 (Figure 5b and Figure S8), V3 being differentially constrained in the CCR5 transmembrane cavity (mean RMSD of V3 Cα atoms = 1.29 ± 0.39Å within system and = 1.89 ± 0.34Å between systems). In all four complexes, two ionic bonds are formed, between conserved residues of gp120 and CCR5 ECL2 (Arg304/Glu172) and TM (Arg315/Glu283), yet these two interactions are much less frequent in CD4–gp120_#34_–CCR5 (Figure S8). Differences in hydrogen bonding are more important, especially with these two CCR5 residues, Glu172 and Glu283 (Figure 5b,c). Glu172 anchors the V3 stem to ECL2 using a persistent hydrogen bond clamp with Arg304 in CD4–gp120_#25_–CCR5 and CD4–gp120_Bx08_–CCR5, while using two hydrogen bonds with two other residues, Arg305 and Ser306, in CD4–gp120_#34_–CCR5. Glu172 can form hydrogen bond with Arg304, Lys305 and Ser306 in CD4–gp120_JR-FL_–CCR5. In CD4–gp120_#34_–CCR5 and CD4–gp120_JR-FL_–CCR5, the conserved Arg304 is also able to interact with another residue of ECL2, Thr177. Noteworthy, positions 305 and 306 show unique sequence variation (Arg305 specific to gp120_#34_ and Gly306 specific to gp120_#25_). Interactions made between the V3 tip and Glu283 in the major subpocket of CCR5 are also a hallmark of gp120. Glu283 carboxylate forms a single hydrogen bond with Arg315 guanidinium in CD4–gp120_Bx08_–CCR5 and CD4–gp120_JR-FL_–CCR5, two in CD4–gp120_#25_–CCR5, an additional hydrogen bond with Gly314 or Arg315 backbone in CD4–gp120_Bx08_–CCR5 and else only hydrogen bonds with Gly314 or the Arg315 backbone in CD4–gp120_#34_–CCR5. Overall, changes in the binding mode of the conserved Arg315 correspond to subtle yet significant conformational adaption of the various gp120s to the major subpocket.

In conclusion, our simulation data clearly illustrate that the networks of interactions between gp120s and CRS2 vary as a function of the nature of the V3 tip, thereby differentially shaping the conformation of the binding pocket of CCR5. This could propagate different conformational rearrangements along the receptor and explain why binding of the different gp120s translates into conformational differences at the cytoplasmic side of the receptor.

### 3.6. Mutations in CRS2 Differentially Influence the Binding of Distinct gp120s to CCR5

**Figure 6:**
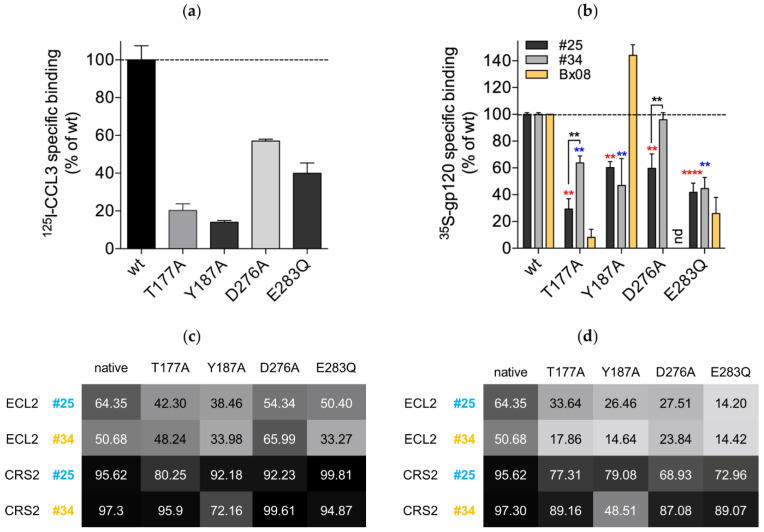
Mutations in CCR5′s CRS2 differentially influence the binding of different gp120s: (**a**) specific binding of ^125^I-CCL3 (0.2 nM) to membranes from HEK 293T cells expressing similar levels of wild-type (wt) CCR5 or the indicated CCR5 mutants. Specific binding was determined by subtracting from total binding the non-specific binding determined on wt-CCR5-expressing membranes in the presence of the CCR5 inverse agonist maraviroc at 10 μM. Results are expressed relative to binding to the wild-type receptor. They represent means ± SEM of two independent experiments performed in duplicate; (**b**) specific binding of ^35^S-labeled gp120_#25_, gp120_#34_ or gp120_Bx08_ (10 nM) in the presence of excess sCD4 (400 nM) to wt-CCR5 or CCR5 mutants. Results for gp120_Bx08_ are from reference [58]. Results for gp120_#25_ and gp120_#34_ are from three independent experiments carried out in duplicate. Specific binding of the glycoproteins was determined as described above in panel (**a**). Results, which are expressed relative to wild-type CCR5, represent means ± SEM. nd: not determined. ** *p* < 0.01, **** *p* < 0.0001 using the Mann–Whitney U-test. Statistics in red and blue are relative to binding to wt-CCR5 for gp120_#25_ and gp120_#34_, respectively; (**c**) percent of common positions of ECL2 and CRS2 during the simulations in wild-type CCR5 (native) or the indicated CCR5 mutants bound to gp120_#25_ or gp120_#34_; (**d**) percent of common positions of ECL2 and CRS2 during the simulations between native and mutated CCR5 bound to gp120_#25_ or gp120_#34_; (**c**,**d**). The proportion of common conformations is computed from the all-against-all comparison of one in ten snapshots issued from the simulation of the same complex (**c**) or the systematic pairwise comparison of one in ten snapshots issued during the simulation of the native and mutated complexes (**d**). Position is common to two snapshots if the deviation of the Cα coordinates of the domain is low (RMSD < 2 Å).

Our simulations predict that distinct gp120s position differently in the CRS2 of CCR5. To further explore this issue, we mutated CCR5 in silico at four key positions: one in ECL2, Thr177, one, Tyr187, in the extracellular part of TM5 and two, Asp276 and Glu283, in the transmembrane cavity. We previously showed that the binding of gp120_Bx08_ to CCR5 is differentially sensitive to mutations T177A, Y187A and E283Q [58]. Here, we extended our investigations to gp120_#25_ and gp120_#34_, and to the effect of the D276A mutation on their binding to CCR5 (Figure 6). 

In the simulated structures, Thr177, Tyr187, Asp276 and Glu283 form interaction networks with V3 and/or other regions of CCR5 that differ between CD4–gp120_#25_–CCR5, CD4–gp120_#34_–CCR5 and CD4–gp120_Bx08_–CCR5 (Table 1). The Thr177 side chain frequently interacts with another residue of ECL2 in CD4–gp120_Bx08_–CCR5 and somewhat less in CD4–gp120_#25_–CCR5 (hydrogen bond with Glu172), while it interacts with V3 in CD4–gp120_#34_–CCR5 (hydrogen bond with Arg304). The Tyr187 side chain contributes to ECL2 structuring in CD4–gp120_#25_–CCR5 (hydrogen bond with Ser180 and π-stacking with Phe182), where it also bridges ECL2 from V3 (hydrogen bond with Ile307). In contrast, there are practically no intramolecular or intermolecular interactions with this residue in CD4–gp120_#34_–CCR5, and very few in CD4–gp120_Bx08_–CCR5. The Asp276 side chain mainly interacts with Thr319 of V3 and with Asn258 of TM6 in CD4–gp120_#25_–CCR5, while these two interactions are reduced in favor of contacts with Lys22 of the CCR5 N-terminal domain in CD4–gp120_#34_–CCR5 and even more in CD4–gp120_Bx08_–CCR5. Lastly, as described above, Glu283 is differentially engaged in networks of hydrogen and ionic bonds with Gly314 and Arg315 of the V3 tip in the four studied complexes. Due to its central position in the 7-TMs, Glu283 also forms intramolecular interactions (with Tyr108 in TM3, Tyr251 in TM6 and Gln280 in TM7), according to gp120-specific patterns.

To investigate the effects of the mutations on gp120 binding, we expressed wild-type CCR5 or either of the different CCR5 mutants in HEK 293T cells. The CCR5 mutants were all expressed similarly to the wild-type receptor, as determined by flow cytometry analysis using the anti-CCR5 mAbs 2D7, 45531 and CTC5, which target the proximal and distal part of ECL2 and the N-terminal domain, respectively. As a control, we also performed binding experiments of the natural CCR5 ligand chemokine ^125^I-CCL3 (Figure 6a). As similarly seen in our previous work [58], mutations T177A, Y187A and E283Q strongly decrease chemokine binding. The D276A substitution also decreases the binding of ^125^I-CCL3, as reported [59], but less dramatically.

We next performed binding experiments of gp120s in complex with sCD4 to different receptors. As expected from our simulations, all mutations differentially influence the binding of gp120s (Figure 6b). The T177A mutation almost cancels the binding of gp120_Bx08_, and significantly decreases that of gp120_#25_, but much less that of gp120_#34_. This finding suggests that gp120_#25_ and even more gp120_Bx08_ are very sensitive to Thr177-dependent changes in CCR5 structure and dynamics, while gp120_#34_ can adapt quite easily to loss of hydrogen bonding between the base of its V3 loop and Thr177. The MD simulation of the mutated CCR5 in complex with gp120_#25_ and gp120_#34_ shows limited ECL2 repositioning in response to the T177A mutation, but a strong indirect effect on ECL3 (Figure S9). Besides, the binding mode of either of the gp120_#25_ and gp120_#34_ to CCR5 is largely modified by the T177A mutation, with effects observed not only in the close vicinity of the mutated residue, yet in the whole CRS2. Simulations differentiate CD4–gp120_#25_–[T177A]CCR5 and CD4–gp120_#34_–[T177A]CCR5 with a more important increase of the flexibility of CRS2 in the first one (Figure 6c) and larger differences in ECL2 conformation in the second one (Figure 6d). These increased changes in CRS2 flexibility may explain why gp120_#25_ binding is decreased more than gp120_#34_ binding in [T177A]CCR5, compared with the wild-type receptor. 

The Y187A mutation inhibits gp120_#25_ and gp120_#34_ binding by 40–50% compared with wild-type CCR5, while our previous results showed that it preserves, and even slightly increases, gp120_Bx08_ binding [58]. In previous work, it was also shown that the mutation does not influence entry of JR-FL [60], suggesting that it also does not influence binding of gp120_JR-FL_. Our results suggest that the hydrogen bond between Tyr187 and Ile307 of V3 is not critical for binding of the glycoproteins gp120_#25_ and gp120_JR-FL_. However, Tyr187 may indirectly regulate gp120 binding, possibly through stabilization of particular conformations of the receptor (Figure 6c,d). The MD simulation of CD4–gp120_#25_–[Y187A]CCR5 and CD4–gp120_#34_–[Y187A]CCR5 shows again that the mutation affects ECL3 positioning and largely modifies the network of interactions between the gp120 and CRS2. We also observe that the Y187A mutation increases the flexibility of ECL2 in the two complexes, but that of CRS2 only in the second one (Figure 6c). 

The effects of mutations D276A and E283Q further highlight that different gp120s have different structural requirements for binding CCR5. The D276A mutation decreases the binding of gp120_#25_ yet does not affect the binding of gp120_#34_. The side chain of Asp276 is constrained in simulated CD4–gp120_#25_–CCR5, with two persistent hydrogen bonds formed with V3, while it oscillates between several binding partners in simulated CD4–gp120_#34_–CCR5, thereby suggesting its mutation to Ala may directly impact the binding of gp120_#25_. In agreement with this, the simulations show that the D276A mutation affects the conformation of CRS2 more in the complex with gp120_#25_ than in the complex with gp120_#34_ (Figure 6d).

The binding of gp120_#25_, gp120_#34_ as well as gp120_Bx08_ is inhibited by the E283Q mutation. Mutation of Glu283 has also been shown to prevent the binding of gp120 from the Yu2 strain [11]. These results illustrate the major role played by the interaction network between Glu283 and the tip of V3 in the binding of gp120s. Furthermore, the E283Q mutation replaces a negatively charged carboxylate with a neutral amide in the central part of the CCR5 transmembrane cavity, which translates into allosteric effects in ECL2 and CRS2, as well as changes in the binding mode of the gp120_#25_ and gp120_#34_. CRS2 experiences subtle structural adaptation (more pronounced for gp120_#25_, Figure 6d); and the dynamics of distant regions of the receptor are affected, for example ECL2 flexibility is increased (more pronounced for gp120_#34_, Figure 6c).

## 4. Discussion

In a previous work, we showed that distinct HIV-1 gp120s exhibit divergent binding levels to CCR5 on cell lines and primary cells [29,30]. Similar results were obtained with other ligands of the receptor, e.g., mAbs [30,31,32,33] and chemokines [35,38]. One explanation for these results is that these ligands bind differentially to heterogeneous forms of the receptor, which exist in different proportions at the cell membrane. HIV-1 itself uses particular CCR5 molecules for entry into target cells [29,30] and escape inhibition by CCR5 chemokines [20,38] and small molecule inhibitors [61]. Several mechanisms have been put forward to contribute to CCR5’s diversity and influence its ligand binding properties, including differences in post-translational modifications [35,54], degree of oligomerization [29,36,39] or conformational state [20,31,37]. Computational approaches also proposed that CCR5 exists in different low-energy conformations, each of which is differentially stabilized by structurally distinct entry inhibitors [62]. However, it remains poorly known whether distinct HIV-1 gp120s/strains recognize/stabilize distinct CCR5 conformations and, if so, what the natures of these conformations are.

To address these issues, we carried out MD simulations of CCR5 in its free form or bound to four different gp120s in complex with sCD4, taking as template the recently published cryo-EM structure of the CD4–gp120_92BR020_–CCR5 complex [17]. Our results emphasize that the free receptor exhibits a continuum of different conformations. Some domains are highly mobile during the simulation time, in particular the N-terminal domain (Figure 2b), ECL3, ICL2 and ICL3 (Figure 3, Figures S4a and S6), while the 7-TM helices are much more rigid (Figure 3, Figures S3, S4a and S6). The receptor loops are also highly labile in the gp120-bound complexes, although they are oriented differently, compared to the free receptor (Figure 3, Figures S6 and S7). These differences are more marked for ECL2, ECL3, ICL2 and ICL3 (Figure 3 and Figure S6). The positions of the extracellular and intracellular ends of CCR5 TMs also vary upon binding of gp120s, in particular the extracellular ends of TM1, 5, 6 and 7, and the intracellular ends of TM5, and to a lesser extent of TM6 and 7 (Figure 4). On the whole, the conformations adopted by the four gp120-bound receptors are closer to each other than between any of them and free CCR5.

The simulations revealed that the four CD4–gp120–CCR5 complexes sample different, yet partly similar, conformational ensembles. The four gp120s differentially influence orientations of ECL2, ECL3 and ICL3 of CCR5 (Figure 3 and Figure S6) as well as the relative positions of the extracellular and intracellular ends of the receptor TMs (Figure 4). On the whole, the four conformational populations of CCR5 are as different from each other as from the cryo-EM structure of CD4–gp120_92BR020_–CCR5 [17] (PDB ID: 6MEO, resolution: 3.90 Å) or from the X-ray structure of the complex between CCR5 and the entry inhibitor maraviroc [19] (PDB ID: 4MBS, resolution: 2.71 Å) (Tables S2 and S3). However, it should be noted that during the relatively short simulation time we used (100 ns), the amplitude of motion experienced by the proteins remained low. The maximal average RMSD calculated on the most flexible region of CCR5 (ECL3) between two different complexes is 7.04 Å (Figure S6). The conformational states sampled by our trajectories actually correspond to fast dynamic phenomena, without important energy barrier. They therefore describe a subset of the whole conformational landscape accessible and observable by biophysical techniques such as NMR, which are better suited to describe larger conformational movements of longer duration [63]. Our simulations, however, suggest that subtle conformational changes of CCR5 may have functional consequences. For instance, the four gp120s influence differently the conformations of extracellular domains of CCR5 (Figure 4 and Figure S7) and, in so doing, accessibility to CRS2. Therefore, even small differences in the conformation of the extracellular domains of CCR5 may differentially impact the binding of different gp120s. Under steady-state conditions, we previously showed that the four gp120s used here label different amounts of CCR5 in cellular membranes [29]. This suggests that in a cellular context, some conformations of CCR5, which are differentially bound by the four gp120s, may not be in equilibrium with each other.

The five CCR5 systems described in the present study, although conformationally distinct and highly dynamic, are all characteristic of an inactive state of a class A GPCR that is not competent to bind a G protein or other intracellular partners (e.g., arrestins) [64]. Indeed, the activation of the receptor involves an outward displacement of TM6, which opens a cavity at its intracellular side for G protein coupling [65]. In all our simulations, TM6 remains in a position characteristic of the inactive state. The gp120s here, however, induce distinctive changes in the receptor that may be predictive of ampler conformational rearrangements towards its activation. Compared to free CCR5, the receptor bound to gp120 shows reorientations of the intracellular ends of TM5, TM6 and TM7, which are the three regions in class A GPCRs whose concerted movements upon agonist binding allow the outward movement of TM6 and coupling to G protein. In the prototypic class A GPCR β2 adrenergic receptor (β2AR), stabilization of an active state involves key interactions along the TM5–TM7 interface that act as microswitches connecting the orthosteric binding site to the G protein binding region [66]. In our simulations of CD4–gp120–CCR5, TM5 adopts a strict canonical α-helical structure for two-thirds of its length on the intracellular side, except for the residues Met210–Ile212, Pro206, which are conserved in the GPCR family, and Gly202, whose position corresponds to the TM5 bulge of the β2AR. In β2AR, the TM5 bulge represents the most proximal microswitch and its structural rearrangement is highly correlated with agonist efficacy [66]. In our simulations of CD4–gp120–CCR5, the gp120s also induce a characteristic bending in TM6. Again, simulations spot in CCR5 the known microswitches of GPCRs. Here, the sequence 247–250, including the toggle switch Trp248 and the conserved Pro250, modulates the bending of a strict canonical portion of the α-helix. The most distal of these microswitches in β2AR and most of class A GPCR is the “ionic lock”, established between the arginine of the conserved “DRY” motif at the cytoplasmic end of TM3 (Arg3.50) and an acidic residue at the cytoplasmic end of TM6 (Asp/Glu6.30). This ionic lock is present in the inactive state and is broken when the receptor is activated, contributing to the outward movement of TM6. Interestingly, this ionic lock cannot exist in CCR5, where a positively charged arginine is present in TM6 instead of the conserved Asp/Glu, explaining that CCR5 is endowed with constitutive activity [67]. In the absence of this ionic lock, we anticipate that CCR5 bound to gp120 could more readily evolve into signaling-competent conformations.

A large number of studies have reported the ability of HIV-1 Envs to trigger CCR5 signaling by a variety of approaches, ranging from the analysis of individual signaling pathways of GPCRs (e.g., calcium mobilization) to the collective analysis of cell responses (e.g., transcriptomic analysis) (for reviews see references [21,22]). However, the underlying molecular mechanisms remain largely unknown. Many of these studies have focused on laboratory-adapted Envs, including JR-FL Env, described here, implicitly assuming that the findings could be generalized to other Envs [25,68,69,70,71,72]. Our MD simulations and mutagenesis data, however, suggest that other Envs, including primary Envs that are characterized by huge sequence variability [73,74,75], may exhibit distinct signaling properties. Indeed, the four gp120s studied here differentially shape the CCR5 transmembrane cavity, a process that may translate into distinct patterns of receptor activation [18,66]. The gp120s also differ in that they reorient differently the intracellular ends of TM5–7 (Figure 4) and even more strikingly ICL3 (Figure 3, Figures S6e and S7). These data suggest that the gp120s can rearrange the intracellular cavity of CCR5 differentially, which could lead to differences in the nature of the intracellular partners with which the receptor interacts [65]. In fact, biased agonism or functional selectivity has already been reported for a wide variety of CCR5 ligands [76,77,78,79], and our data strongly suggest that HIV-1 Envs are no exception to this rule. If so, an interesting perspective for future work would be one exploring whether the differences in signaling between different Envs contribute to differential shaping of the phenotypic properties of viruses and their role in the pathogenesis of infection. In this respect, the gp120s characterized herein could constitute suitable Env samples.

## 5. Conclusions

In conclusion, simulation data have complemented the cryo-EM structure to picture the networks of interactions in CRS2 as a function of gp120s, thus providing a relationship between sequence variations in V3 and the different conformational populations of CCR5. Conversely, one can speculate that this is the reason why changes in CCR5 conformation differentially influence the binding of different gp120s [29].

## Figures and Tables

**Table 1:** Frequency in the simulated structures of the hydrogen bonds, ionic bonds and π-stacking interactions involving the four CCR5 mutated residues. Interaction frequency values greater than 50% are in bold.

CCR5 Mutated Residue			Interaction Type	Interacting Residue			gp120_#25_	gp120_#34_	gp120_Bx08_	gp120_JR-FL_
Region	Residue	Group		Region	Residue	Group				
ECL2	Thr177	OH	hydrogen bond	V3	Arg304	C(NH_2_)_2_^+^	0%	**75%**	2%	**57%**
				ECL2	Glu172	COO^−^	26%	<1%	**66%**	16%
TM5	Tyr187	OH	hydrogen bond	V3	Ile307	NH	**82%**	0%	8%	25%
				ECL2	Ser180	CO	**87%**	<1%	3%	26%
		Phenyl ring	π-stacking	TM5	Phe182	phenyl ring	15%	<1%	4%	4%
TM7	Asp276	COO^−^	ionic bond	N-ter	Lys22	NH_3_^+^	4%	32%	**60%**	**51%**
			hydrogen bond	V3	Thr319	OH	**82%**	46%	**82%**	1%
				TM6	Asn258	NH_2_	**66%**	10%	8%	32%
				N-ter	Lys22	NH_3_^+^	<1%	26%	**54%**	48%
				TM6	Gln261	NH_2_	9%	<1%	2%	33%
TM7	Glu283	COO^−^	ionic bond	V3	Arg315	C(NH_2_)_2_^+^	**100%**	25%	**99%**	**100%**
		COO^−^	hydrogen bond	V3	Gly314	NH	**65%**	**94%**	**98%**	0%
				V3	Arg315	C(NH_2_)_2_^+^	**100%**	25%	**≈** **100%**	**≈** **100%**
				V3	Arg315	NH	<1%	35%	**97%**	0%
				TM3	Tyr108	OH	24%	5%	<1%	**99%**
				TM6	Tyr251	OH	**97%**	8%	**75%**	**58%**
				TM7	Gln280	NH_2_	**67%**	40%	<1%	16%

## Data Availability

Preparation scripts and simulations are accessible via an online repository (https://seafile.unistra.fr/d/6bf36e02ac2449669305/).

## Supplementary Materials

The following are available online at https://www.mdpi.com/article/10.3390/v13071395/s1. Table S1: Restraints applied during heating and equilibration, Figure S1: Sequences and mutations of gp120 of the four HIV-1 strains used in this study as well as of HxB2 and 92BR020 strains, Figure S2: Cryo-EM structure of the CD4–gp120–CCR5 complex, Figure S3: Time series of root mean square deviation (RMSD) values of Cα atoms using as reference the input coordinates, Figure S4: Flexible domains in CD4, gp120 and CCR5, Figure S5: Principal component analysis (PCA) of CCR5_20-313_, Figure S6: Average deviation in angstrom of coordinates of the CCR5 loops and 7-TMs, Figure S7: Position of CCR5 extracellular loops, Figure S8: Ionic interactions in CCR5/g120 interfaces, Table S2: Percent of common positions of coordinates of the CCR5 loops and 7-TMs, Table S3: Average deviation in angstrom of coordinates of the CCR5 loops and 7-TMs, Figure S9: Position of wild-type and mutated CCR5 extracellular loops in CD4–gp120_#25_–CCR5 and CD4–gp120_#34_–CCR5 complexes.
