# Supplementary material for: Modeling of CCR5 Recognition by HIV-1 gp120: How the Viral Protein Exploits the Conformational Plasticity of the Coreceptor
Source: Viruses. 2021 Jul 18;13(7):1395. doi: 10.3390/v13071395 (PMC8310383; doi:10.3390/v13071395)
Supplement: Supplementary file 1 [file viruses-13-01395-s001.zip › viruses-1243963-supplementary.pdf]

**Table S1.** Restraints applied during heating and equilibration.

| Time (ps) | Restraint (kcal/mol$^2$) | | | | temperature (K) | |
|---|---|---|---|---|---|---|
| | water | ions | lipids | proteins | Initial | Final |
| 25 | 2.5 | 10 | 2.5 | 10 | 0 | 100 |
| 25 | 2.5 | 10 | 2.5 | 10 | 100 | 200 |
| 25 | 2.5 | 10 | 2.5 | 10 | 200 | 300 |
| 25 | 2.5 | | 2.5 | 5 | 300 | 300 |
| 25 | 1.0 | | 1.0 | 2.5 | 300 | 300 |
| 25 | 0.5 | | 0.5 | 1.0 | 300 | 300 |
| 25 | 0.1 | | 0.1 | 1.0 | 300 | 300 |
| 25 | | | | 1.0 | 300 | 300 |
| 25 | | | | 0.5 | 300 | 300 |
| 25 | | | | | 300 | 300 |

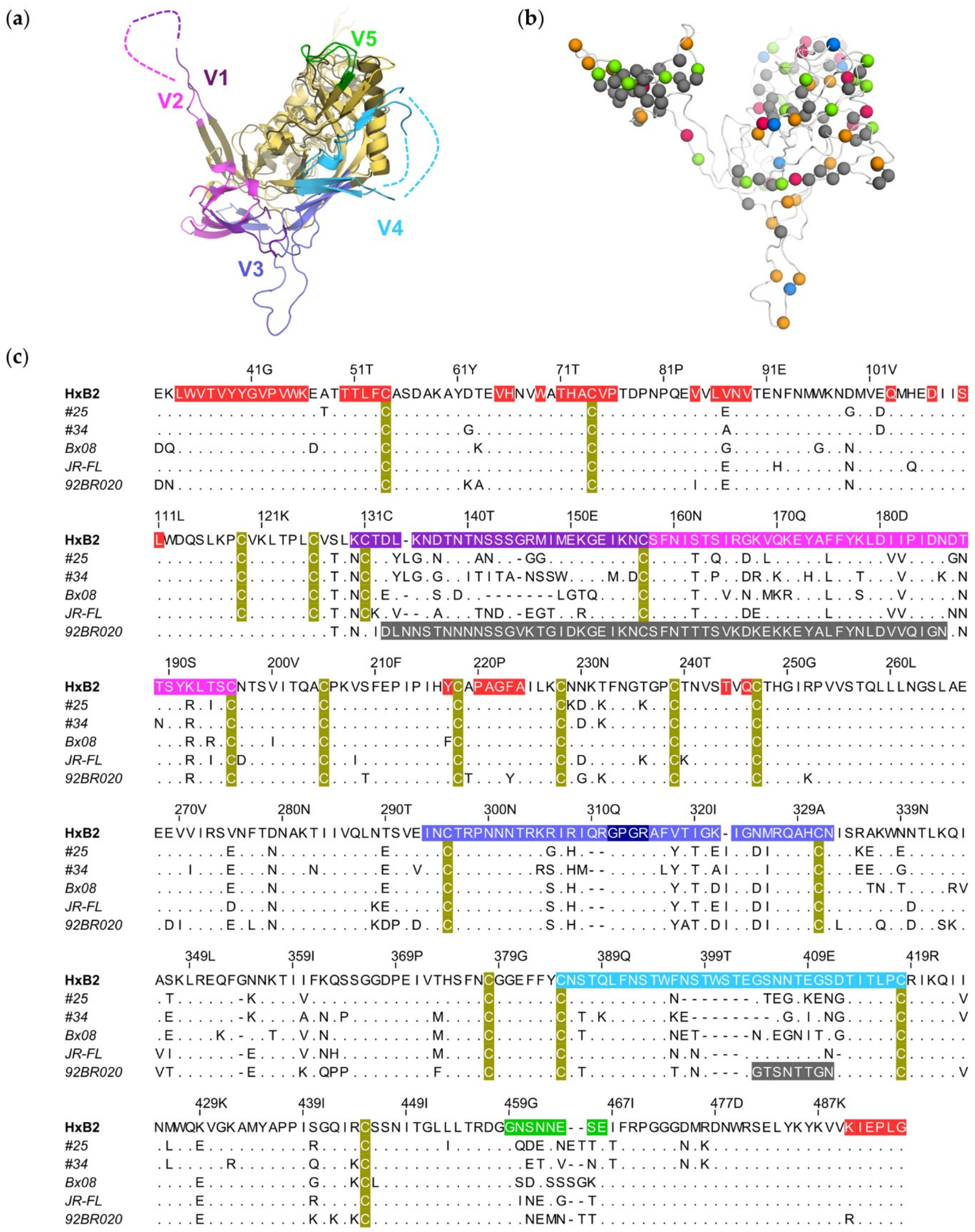


**Figure S1.** Sequences and mutations of gp120 of the four HIV-1 strains used in this study as well as of HxB2 and 92BR020 strains: (**a**) Superimposed structures of closed (gold cartoon, PDB ID: 5CEZ) and open (ocher cartoon, PDB ID: 6MEO) forms of gp120. The gp120 variable loops are highlighted with specific colors: V1 in purple, V2 in mauve, V3 in blue, V4 in cyan and V5 in green. Missing parts

of gp120 in structures are represented by dashed line; (**b**) Open gp120 structure (grey cartoon) highlighting mutated positions (spheres) in the sequence of the variants of #25, #34, Bx08 and JR-FL. Specific positions in gp120#25, gp120#34, gp120Bx08 or gp120JR-FL are colored in blue, orange, green or red, respectively. When a point position is common to two or more gp120s, it is colored in grey; (**c**) Multiple sequence alignment of gp120. The top sequence HxB2 is the standard for gp120 residue sequence and numbering. Only insertion, deletion and variations are shown for the other gp120 sequences, with the exception of the cystein residues in disulfide bridges (dark yellow background). The sequences studied here are #25, #34, bx08 and JR-FL. The bottom sequence 92BR020 is taken from the cryoEM template structure (PDB ID: 6MEO). The gp120 loops are highlighted on the top sequence with the same color as in panel (**a**) (note that the V3 tip is indicated using a darker color). The binding site of gp41, as identified in the *env* closed form (PDB ID: 5CEZ) is highlighted in red.

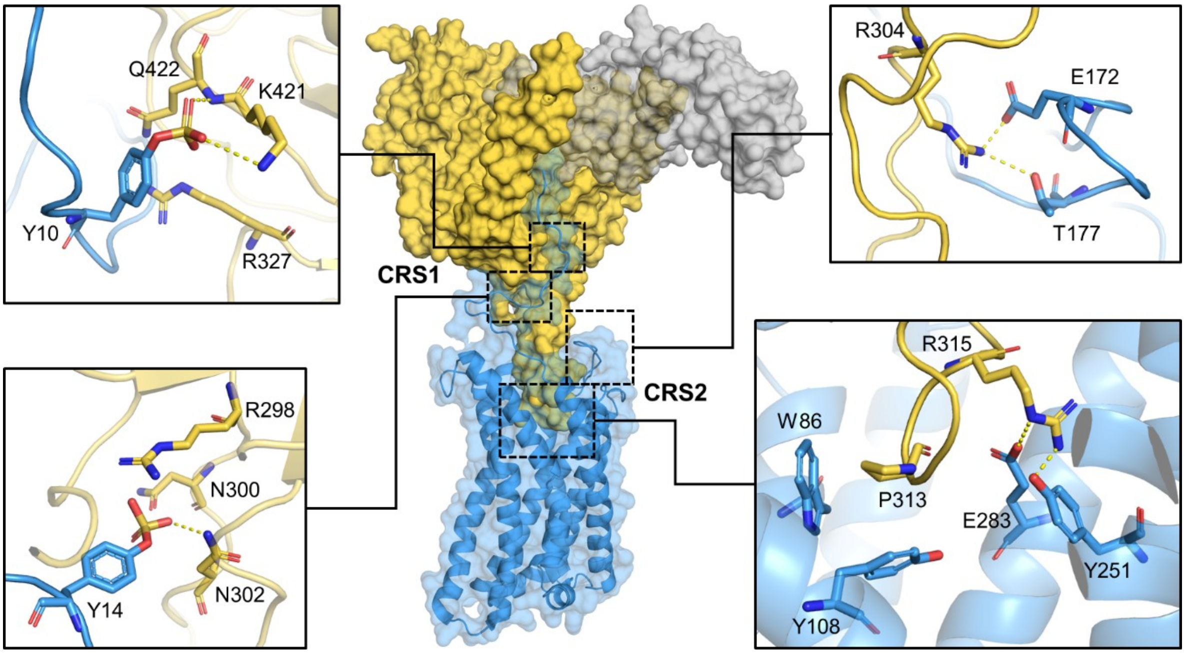


**Figure S2.** Cryo-EM structure of the CD4-–gp120–CCR5 complex (PDB: 6MEO).

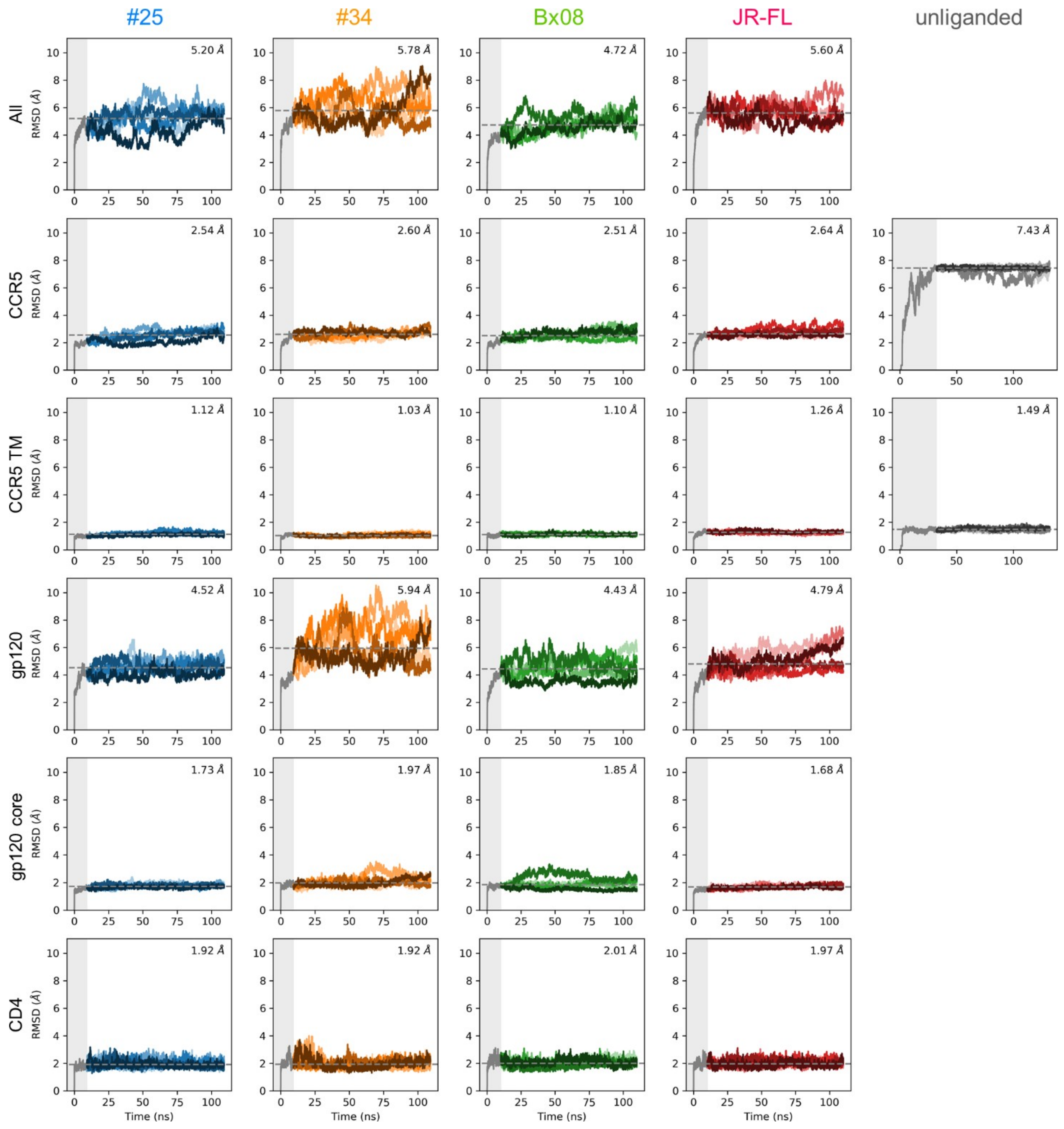


**Figure S3.** Time series of root mean square deviation (RMSD) values of Cα atoms using as reference the input coordinates. RMSD were computed on the full CCR5–gp120–CD4 complex ("All"), the three proteins considered separately ("CCR5", "gp120" and "CD4"), the CCR5 7-TMs ("TM") and the gp120 without the variables loops ("gp120 core"). Data points are colored in grey in the equilibration phase, and in blue, orange, green, red and grey in the production phase of CCR5–gp120$_{\#25}$–CD4, CCR5–gp120$_{\#34}$–CD4, CCR5–gp120$_{Bx08}$–CD4, CCR5–gp120$_{JR\text{-}FL}$–CD4 and unliganded CCR5, respectively. The five independent runs are distinguished by the color tone. The median RMSD of the five runs over the production phase is indicated in plot.

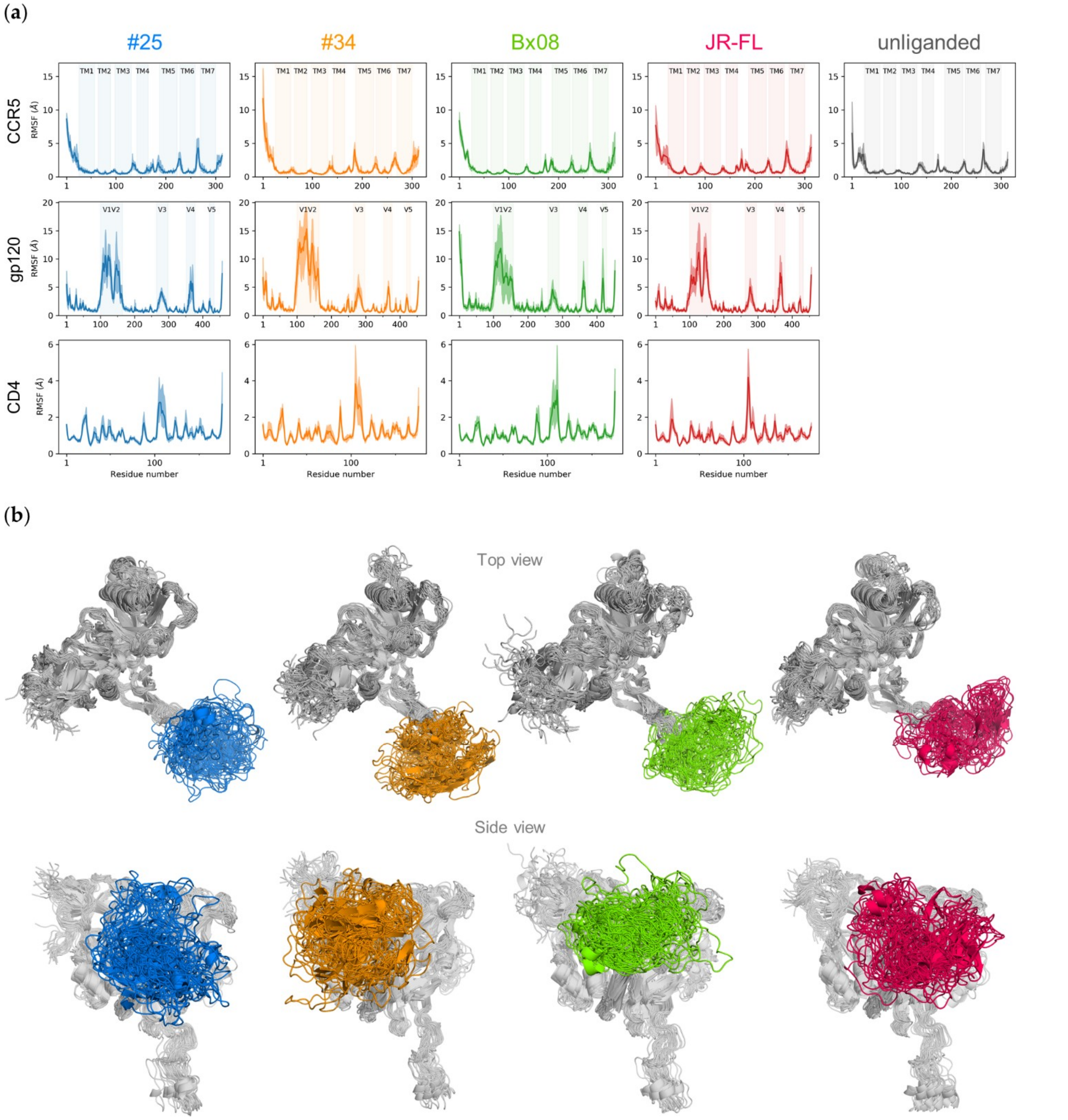


**Figure S4.** Flexible domains in CD4, gp120 and CCR5: (**a**) Fluctuation of the C$\alpha$ atoms of CCR5, gp120 and CD4 over the production phase. All structures were 3D-aligned on the input coordinates; (**b**) Sampling of gp120 structures throughout trajectory. A total of 15 representative structures was selected for each variant. CCR5 and CD4 were not represented. The gp120 main chain is shown as grey wire, except the variable loops V1 and V2 which are colored according to the variants. A point of view from above the lipid membrane is shown first, a point of view in the plane of the lipid membrane.

**(a)**

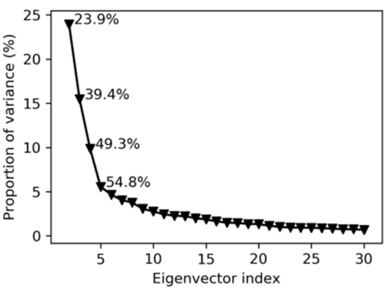

23.9%
39.4%
49.3%
54.8%
Proportion of variance (%)
Eigenvector index


**(b)**

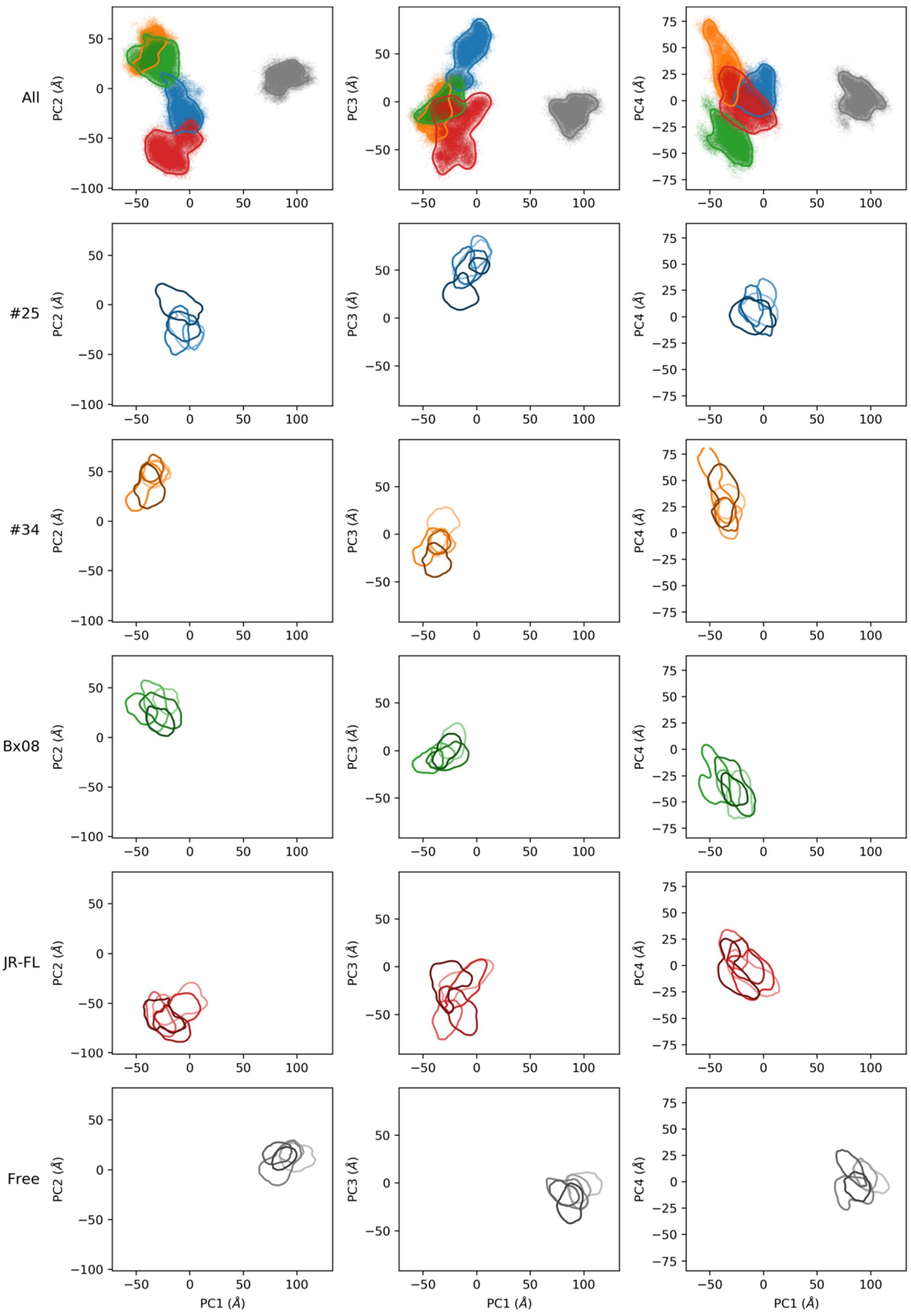

All
#25
#34
Bx08
JR-FL
Free
PC2 (Å)
PC3 (Å)
PC4 (Å)
PC1 (Å)

**(c)**

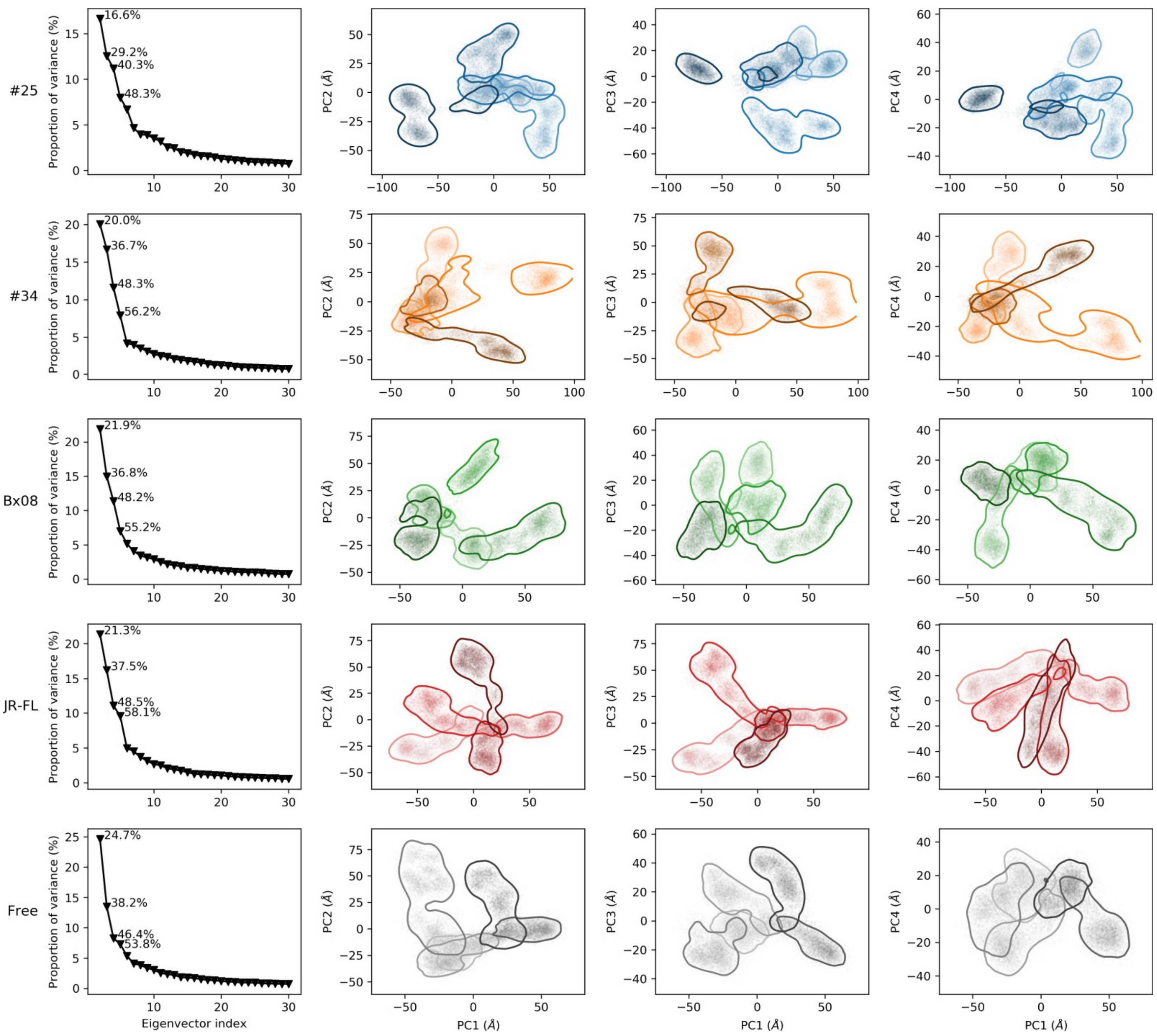


**Figure S5.** Principal component analysis (PCA) of CCR5$_{20\text{-}313}$: **(a)** Contribution to the variance of the eigenvectors. Each triangle represents the eigenvalue associated to an eigenvector and the percentages displayed correspond to the cumulative sum at index N. During the PCA, all trajectories were pooled; **(b)** Projections of molecular dynamics trajectories onto the principal planes defined by the four most significant principal components. From top to bottom: all trajectories; five trajectories of gp120$_{\#25}$-CD4-CCR5; five trajectories of gp120$_{\#34}$-CD4-CCR5; five trajectories of gp120$_{Bx08}$-CD4-CCR5; five trajectories of gp120$_{JR\text{-}FL}$-CD4-CCR5; five trajectories of CCR5. During the PCA, all trajectories were pooled; (**c**) Contribution to the variance of the eigenvectors and projections of molecular dynamics trajectories onto the principal planes defined by the four most significant principal components considering each system separately during the PCA. From top to bottom: five trajectories of gp120$_{\#25}$-CD4-CCR5; five trajectories of gp120$_{\#34}$-CD4-CCR5; five trajectories of gp120$_{Bx08}$-CD4-CCR5; five trajectories of gp120$_{JR\text{-}FL}$-CD4-CCR5; five trajectories of CCR5; Data are colored according to the gp120 (gp120$_{\#25}$: blue; gp120$_{\#34}$: orange; gp120$_{Bx08}$: green; gp120$_{JR\text{-}FL}$: red; no gp120: grey).

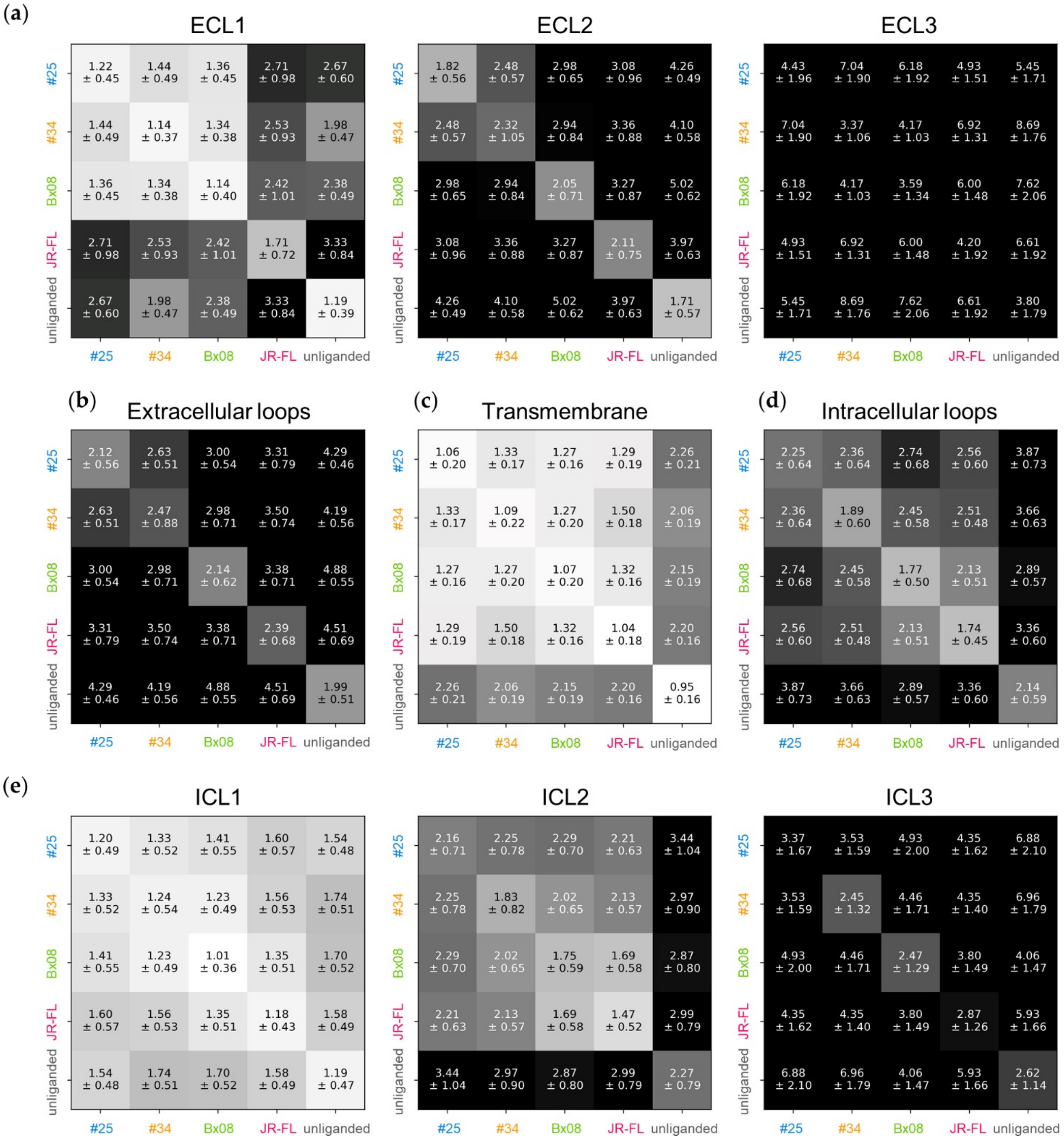


**Figure S6.** Average deviation in angstrom of coordinates of the CCR5 loops and 7-TMs: (**a**) ECL1, ECL2 and ECL3; (**b**) The three ECLs; (**c**) 7-TMs; (**d**) The three ICLs; (**e**) ICL1, ICL2 and ICL3; (**a**)-(**e**) All structures were 3D-aligned on the input coordinates of CCR5 TM. The RMSD of C$\alpha$ atoms is computed from the all-against–all comparison of the snapshots issued from the simulation of the same system (diagonal of the matrix) or the systematic pairwise comparison of the snapshots issued during the simulation of two different systems (off-diagonal terms of the matrix).

(**a**)

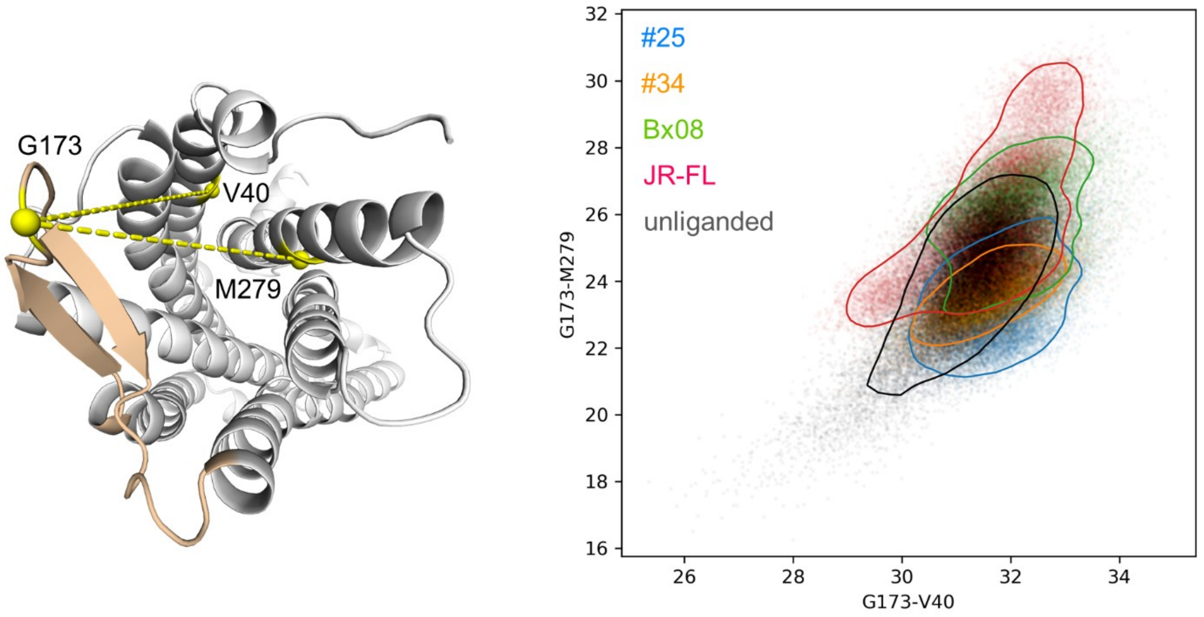


(**b**)

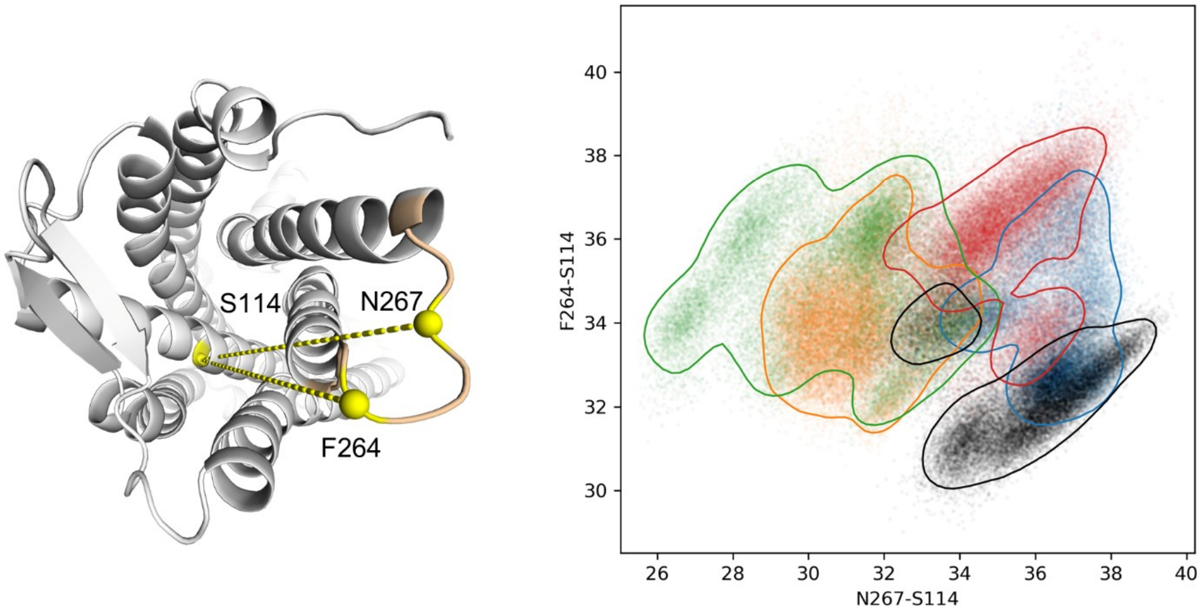


**Figure S7.** Position of CCR5 extracellular loops: (**a**) ECL2; (**b**) ECL3; (**a**)-(**b**) On the left side of the figure, the two distances pairs, which characterize ECL2 or ECL3 position with respect to the seven TMs are shown on the structure of CCR5 viewed from above the plan of the membrane. On the right side of the figure is shown the distribution of the characteristic distance pairs in angstrom over the simulation. Data points of CD4–gp120$_{\#25}$–CCR5, CD4–gp120$_{\#34}$–CCR5, CD4–gp120$_{Bx08}$–CCR5, CD4–gp120$_{JR\text{-}FL}$–CCR5 and free CCR5 are colored in blue, orange, green, red and grey, respectively.

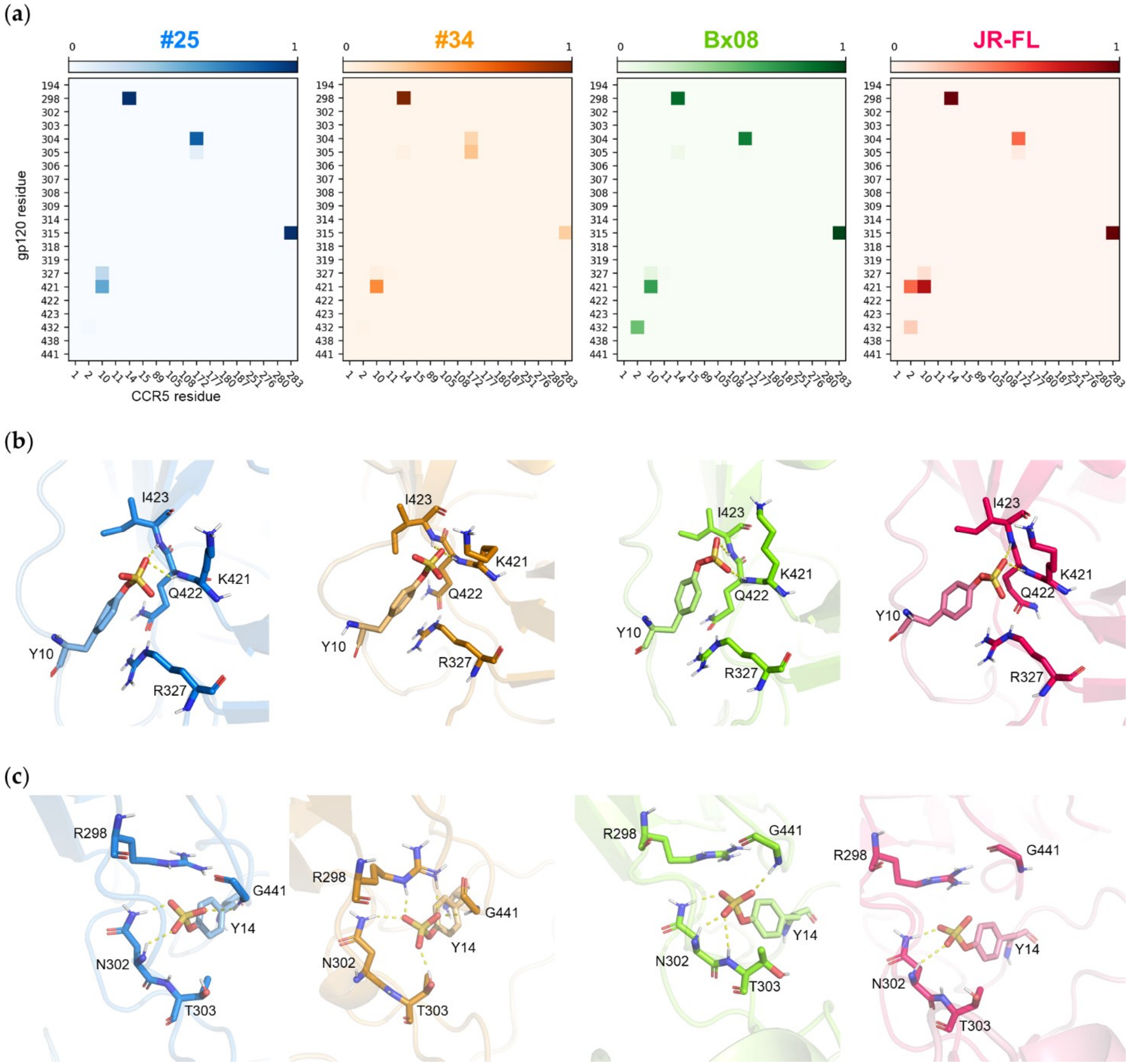


**Figure S8.** Ionic interactions in CCR5/gp120 interfaces: (**a**) Frequency of the ionic bonds formed between CCR5 and gp120 during simulations. Frequency ranges from 0 (light color) to 1 (dark color); (**b**) Key interactions between gp120 bridging sheet and CCR5 Tys10; (**c**) Key interactions between gp120 bridging sheet and CCR5 Tys14; (**b**), (**c**) CCR5 is shown as pale ribbon, residues in interaction as sticks; (**a**)-(**c**) Data points and protein structures of CD4–gp120$_{\#25}$–CCR5, CD4–gp120$_{\#34}$–CCR5, CD4–gp120$_{Bx08}$–CCR5 and CD4–gp120$_{JR\text{-}FL}$–CCR5 are colored in blue, orange, green and red, respectively.

**Table S2.** Percent of common positions of coordinates of the CCR5 loops and 7-TMs. All structures were 3D-aligned on the input coordinates of CCR5 TM. The proportion of common conformations is computed from the comparison of the snapshots issued from the simulation with the cryo-EM structure of CD4–gp120–CCR5 complex (PDB ID: 6MEO) and the RX structure of CCR5–maraviroc complex (PDB ID: 4MBS).

| | | **Transmembrane** | **N-terminal** | **ECL1** | **ECL2 β-hairpin** | **ECL2** | **ECL3** | **ICL1** | **ICL2** | **ICL3** | **Extracellular loops** | **Intracellular loops** |
|---|---|---|---|---|---|---|---|---|---|---|---|---|
| **6MEO** | #25 | 100 | 0.01 | 89.03 | 32.88 | 16.14 | 0.00 | 92.16 | 24.91 | 1.91 | 0.02 | 9.00 |
| | #34 | 100 | 0.00 | 99.32 | 2.98 | 0.98 | 1.24 | 98.19 | 51.28 | 12.99 | 0.73 | 52.34 |
| | Bx08 | 100 | 0.00 | 96.60 | 2.43 | 1.17 | 0.58 | 99.90 | 61.39 | 3.20 | 0.52 | 33.62 |
| | JR-FL | 100 | 0.00 | 28.58 | 5.90 | 0.33 | 0.00 | 91.07 | 75.83 | 0.18 | 0.00 | 31.04 |
| | CCR5 free | 100 | 0.00 | 97.74 | 1.37 | 0.00 | 0.00 | 98.59 | 63.16 | 0.00 | 0.00 | 0.04 |
| **4MBS.A** | #25 | 100 | no data | 8.78 | 1.00 | 0.54 | 0.00 | 98.41 | 60.12 | no data | 0.00 | no data |
| | #34 | 100 | no data | 36.26 | 6.17 | 1.75 | 0.02 | 99.28 | 76.29 | no data | 0.85 | no data |
| | Bx08 | 100 | no data | 42.52 | 34.28 | 8.97 | 0.00 | 100.00 | 84.46 | no data | 0.48 | no data |
| | JR-FL | 100 | no data | 70.39 | 62.34 | 60.28 | 0.00 | 91.86 | 95.16 | no data | 0.00 | no data |
| | CCR5 free | 100 | no data | 84.96 | 29.53 | 0.57 | 0.00 | 99.91 | 20.38 | no data | 0.00 | no data |
| **4MBS.B** | #25 | 100 | no data | 31.38 | 4.47 | 2.78 | 0.00 | 95.20 | 63.19 | no data | 0.00 | no data |
| | #34 | 100 | no data | 69.87 | 22.20 | 6.76 | 0.03 | 96.46 | 80.95 | no data | 3.67 | no data |
| | Bx08 | 100 | no data | 68.94 | 26.39 | 14.97 | 0.00 | 99.94 | 90.91 | no data | 1.58 | no data |
| | JR-FL | 100 | no data | 59.42 | 57.24 | 34.11 | 0.00 | 82.48 | 97.15 | no data | 0.00 | no data |
| | CCR5 free | 100 | no data | 98.22 | 46.87 | 1.42 | 0.00 | 99.38 | 23.54 | no data | 0.00 | no data |

**Table S3.** Average deviation in angstrom of coordinates of the CCR5 loops and 7-TMs. All structures were 3D-aligned on the input coordinates of CCR5 TM. The proportion of common conformations is computed from the comparison of the snapshots issued from the simulation with the cryo-EM structure of CD4–gp120–CCR5 complex (PDB ID: 6MEO) and the RX structure of CCR5–maraviroc complex (PDB ID: 4MBS).

| | | **Transmembrane** | **N-terminal** | **ECL1** | **ECL2 β-hairpin** | **ECL2** | **ECL3** | **ICL1** | **ICL2** | **ICL3** | **Extracellular loops** | **Intracellular loops** |
|---|---|---|---|---|---|---|---|---|---|---|---|---|
| **6MEO** | #25 | 1.18 ± 0.13 | 6.38 ± 1.71 | 1.54 ± 0.37 | 2.26 ± 0.46 | 2.70 ± 0.59 | 6.42 ± 1.83 | 1.31 ± 0.43 | 2.47 ± 0.70 | 4.00 ± 1.14 | 3.67 ± 0.77 | 2.66 ± 0.50 |
| | #34 | 1.01 ± 0.10 | 7.10 ± 1.26 | 1.25 ± 0.26 | 2.47 ± 0.26 | 2.80 ± 0.65 | 3.37 ± 0.67 | 1.19 ± 0.36 | 2.00 ± 0.50 | 2.66 ± 0.61 | 2.82 ± 0.48 | 2.00 ± 0.31 |
| | Bx08 | 1.06 ± 0.08 | 7.49 ± 1.89 | 1.35 ± 0.30 | 3.26 ± 0.64 | 2.99 ± 0.42 | 3.82 ± 0.84 | 1.00 ± 0.26 | 1.88 ± 0.52 | 3.74 ± 1.02 | 3.09 ± 0.43 | 2.24 ± 0.47 |
| | JR-FL | 1.20 ± 0.08 | 7.15 ± 1.72 | 2.59 ± 0.84 | 3.23 ± 1.01 | 3.60 ± 0.83 | 6.08 ± 1.47 | 1.38 ± 0.44 | 1.68 ± 0.49 | 3.60 ± 0.78 | 4.07 ± 0.70 | 2.18 ± 0.33 |
| | CCR5 free | 1.35 ± 0.12 | 24.89 ± 1.03 | 1.40 ± 0.26 | 3.03 ± 0.54 | 3.91 ± 0.40 | 8.39 ± 1.65 | 1.24 ± 0.31 | 1.97 ± 0.53 | 5.96 ± 1.06 | 5.18 ± 0.63 | 3.12 ± 0.39 |
| **4MBS.A** | #25 | 1.20 ± 0.10 | 21.55 ± 1.18 | 2.53 ± 0.40 | 3.22 ± 0.52 | 2.95 ± 0.39 | 6.60 ± 1.87 | 0.92 ± 0.39 | 1.90 ± 0.50 | 6.41 ± 1.05 | 3.82 ± 0.65 | 3.23 ± 0.47 |
| | #34 | 1.08 ± 0.10 | 22.78 ± 1.61 | 2.16 ± 0.44 | 2.80 ± 0.52 | 3.02 ± 0.40 | 3.44 ± 0.67 | 1.10 ± 0.33 | 1.80 ± 0.42 | 5.17 ± 0.56 | 2.95 ± 0.36 | 2.73 ± 0.27 |
| | Bx08 | 1.16 ± 0.09 | 21.01 ± 1.21 | 2.12 ± 0.48 | 2.26 ± 0.52 | 2.73 ± 0.53 | 4.46 ± 0.82 | 0.88 ± 0.24 | 1.51 ± 0.47 | 5.48 ± 0.71 | 2.93 ± 0.40 | 2.74 ± 0.33 |
| | JR-FL | 1.08 ± 0.10 | 20.18 ± 1.26 | 1.67 ± 0.58 | 1.98 ± 0.56 | 1.99 ± 0.46 | 6.56 ± 1.27 | 1.25 ± 0.51 | 1.24 ± 0.41 | 5.90 ± 0.84 | 3.19 ± 0.54 | 2.89 ± 0.34 |
| | CCR5 free | 1.41 ± 0.12 | 19.85 ± 0.44 | 1.61 ± 0.37 | 2.44 ± 0.70 | 2.80 ± 0.40 | 8.42 ± 1.72 | 1.01 ± 0.27 | 2.71 ± 0.77 | 7.53 ± 0.79 | 4.54 ± 0.76 | 3.95 ± 0.35 |
| **4MBS.B** | #25 | 1.18 ± 0.12 | 29.24 ± 1.04 | 2.21 ± 0.41 | 2.81 ± 0.50 | 2.69 ± 0.39 | 6.59 ± 1.87 | 1.11 ± 0.46 | 1.85 ± 0.53 | 6.57 ± 1.08 | 3.65 ± 0.71 | 3.30 ± 0.50 |
| | #34 | 1.01 ± 0.09 | 28.61 ± 1.31 | 1.80 ± 0.40 | 2.39 ± 0.48 | 2.63 ± 0.46 | 3.31 ± 0.61 | 1.00 ± 0.42 | 1.73 ± 0.42 | 5.36 ± 0.58 | 2.65 ± 0.37 | 2.77 ± 0.29 |
| | Bx08 | 1.12 ± 0.08 | 29.64 ± 1.49 | 1.79 ± 0.44 | 2.30 ± 0.47 | 2.45 ± 0.43 | 4.46 ± 0.80 | 0.83 ± 0.26 | 1.37 ± 0.46 | 5.36 ± 0.66 | 2.77 ± 0.36 | 2.65 ± 0.30 |
| | JR-FL | 1.12 ± 0.10 | 29.06 ± 1.18 | 1.97 ± 0.67 | 2.09 ± 0.65 | 2.28 ± 0.56 | 6.60 ± 1.22 | 1.41 ± 0.59 | 1.11 ± 0.39 | 5.92 ± 0.87 | 3.36 ± 0.53 | 2.90 ± 0.36 |
| | CCR5 free | 1.33 ± 0.11 | 19.67 ± 0.59 | 1.28 ± 0.28 | 2.14 ± 0.58 | 2.66 ± 0.35 | 8.30 ± 1.67 | 1.07 ± 0.33 | 2.63 ± 0.75 | 7.29 ± 0.76 | 4.44 ± 0.73 | 3.84 ± 0.35 |

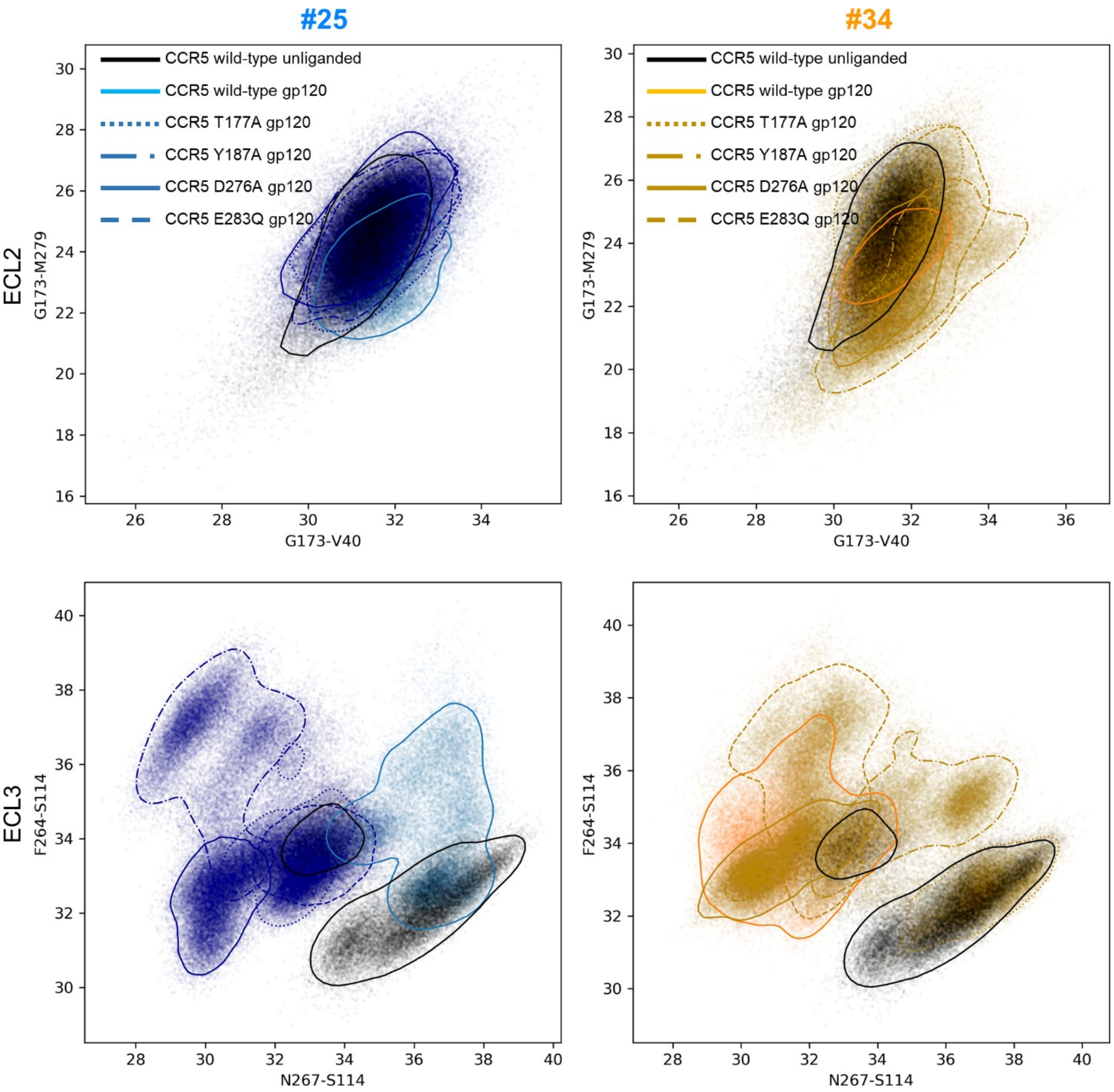


**Figure S9.** Position of wild-type and mutated CCR5 extracellular loops in CD4–gp120#25–CCR5 (left) and CD4–gp120#34–CCR5 (right) complexes: the distribution of the characteristic distance pairs in angstrom over the simulation is shown for ECL2 (top) and ECL3 (bottom).
